# Supplementary material for: Creating complex protocells and prototissues using simple DNA building blocks
Source: Nat Commun. 2023 Mar 10;14:1314. doi: 10.1038/s41467-023-36875-5 (PMC10006096; doi:10.1038/s41467-023-36875-5)
Supplement: Supplementary file 1 — Supplementary Information [file 41467_2023_36875_MOESM1_ESM.pdf]

SUPPORTING INFORMATION

Creating complex protocells and prototissues using simple DNA building blocks

Nishkantha Arulkumaran[1], Mervyn Singer[1], Stefan Howorka[2], Jonathan R. Burns[2*]

[1]Bloomsbury Institute of Intensive Care Medicine, Division of Medicine, University College London, London, WC1E 6BT, United Kingdom

[2]Department of Chemistry, Institute of Structural and Molecular Biology, University College London, London,WC1H 0AJ, United Kingdom

Contents

1 Supporting information 3
1.1 Nanotube and fiber component DNA sequences 3
1.2 Buffers and salt conditions 4
1.3 DNA construct pooled sequences 4
1.4 Dimensions of subunit, nanotubes and fibers 5
1.5 2D map of subunit and joined subunits 6
2 Supplementary data 7
2.1 Structural characterization of DNA nanotubes and fibers 7
2.2 Solution-phase properties and formation yields of DNA macrostructures 10
2.3 CLSM analysis of protocells 12
2.4 Melting profiles of 42 mM $MgCl_2$ DNA fibers 12
2.5 Assembling DNA fibers inside protocells 13
2.6 Determining protocell lipid generalizability 19
2.7 Determining protocell osmolar stability and rupturing properties 19
2.8 Generating multi-DNA cytoskeleton protocells 21
2.9 Compartmentalized proximity control of cholesterol DNA fibers 23
2.10 Magnetic particle coated fibers inside protocells 24
2.11 Vesicle-in-vesicle cytoskeleton supported protocells 25
2.12 DNA fiber exoskeleton GUVs 26
2.13 Determining nuclease resistant properties of exoskeleton protocells 26
2.14 Rupturing properties of dye-loaded vesicles 27
2.15 DNA fibers in high glucose 27
2.16 Prototissue particle tracking analysis 28
2.17 Controlling prototissue formation 29
2.18 Generating multi-layered prototissues 30
2.19 Building prototissues of different sizes 32
2.20 Idenfitying the influence of exoskelelton fibers during prototissue formation 33
2.21 Investigating prototissue packing with different exoskeletons 34
2.22 Prototissue hyperosmolarity assay 35
2.23 Cross-linking prototissues with external DNA fibers 36
2.24 Studying prototissue dynamics 38
2.25 Exploring human blood cell compatiblty and viability 39

1 Supporting information

1.1 Nanotube and fiber component DNA sequences

ID	Sequence (5' → 3')
1	CTCAGTGGACAGCCGTTCTGGAGCGTTGGACGAAACT
2	GTCTGGTAGAGCACCACTGAGAGGTA
3	CCAGAACGGCTGTGGCTAAACAGTAACCGAAGCACCAACGCT
4	CAGACAGTTTCGTGGTCATCGTACCT
5	CGATGACCTGCTTCGGTTACTGTTTAGCCTGCTCTAC
1FAM	/56-FAM/CTCAGTGGACAGCCGTTCTGGAGCGTTGGACGAAACT
1 Chol hair	AGCTGGCGAAAGGGGGATGTG<br>CTCAGTGGACAGCCGTTCTGGAGCGTTGGACGAAACT
1 Biotin	/5BioSG/CTCAGTGGACAGCCGTTCTGGAGCGTTGGACGAAACT
4Cy3	/5Cy3/CAGACAGTTTCGTGGTCATCGTACCT
5Cy5	/5Cy5/CGATGACCTGCTTCGGTTACTGTTTAGCCTGCTCTAC
5A405	/Alexa405/CGATGACCTGCTTCGGTTACTGTTTAGCCTGCTCTAC
Chol	CACATCCCCCTTTCGCCAGCT/3CholTEG/
Chol FAM	/56-FAM/CACATCCCCCTTTCGCCA GCT/3CholTEG/

Table S1. DNA sequences used to generate nanotubes and fibers. Where stated, Alexa405, FAM, Cy3 or Cy5 fluorophore modifications were introduced. The underlined sequence of 1 Chol hair forms a duplex with the Chol strand.

1.2 Buffers and salt conditions

Construct	Media
Oligonucleotides	1x TAE pH 8.3, 0 mM $MgCl_2$
Nanotube	1x TAE pH 8.3, 14 mM $MgCl_2$
28 mM $MgCl_2$ fiber	1x TAE pH 8.3, 28 mM $MgCl_2$
42 mM $MgCl_2$ fiber	1x TAE pH 8.3, 42 mM $MgCl_2$
100 mM $MgCl_2$ fiber	1x TAE pH 8.3, 100 mM $MgCl_2$

Table S2. Different media conditions used to assemble oligonucleotides, nanotubes and fibers.

1.3 DNA construct pooled sequences

Construct	Component strands
Single strand control (ss)	3
Double strand control (ds)	3, 5
DNA nanotube / fiber	1, 2, 3, 4, 5
DNA nanotube / fiber - 1	1, 2, 3, 5
Hairy nanotube / fiber	1 Chol hair, 2, 3, 4, 5
Biotin fiber	1 Biotin, 2, 3, 4, 5

Table S3. DNA strand combinations used to generate native and modified nanotubes or fibers, 1 equivalent of each strand. Where defined in the text, A405, FAM and Cy3 labeled versions of oligonucleotides were substituted for its unmodified equivalent in a molar ratio of 0.1 eq., to generate the cholesterol nanotube and cholesterol fiber, Chol strand was added to the hairy nanotube / fiber after annealing in a 0.1 eq. ratio. Both the cholesterol and dye modifications can alter the macrostructure properties.

1.4 Dimensions of subunit, nanotubes and fibers

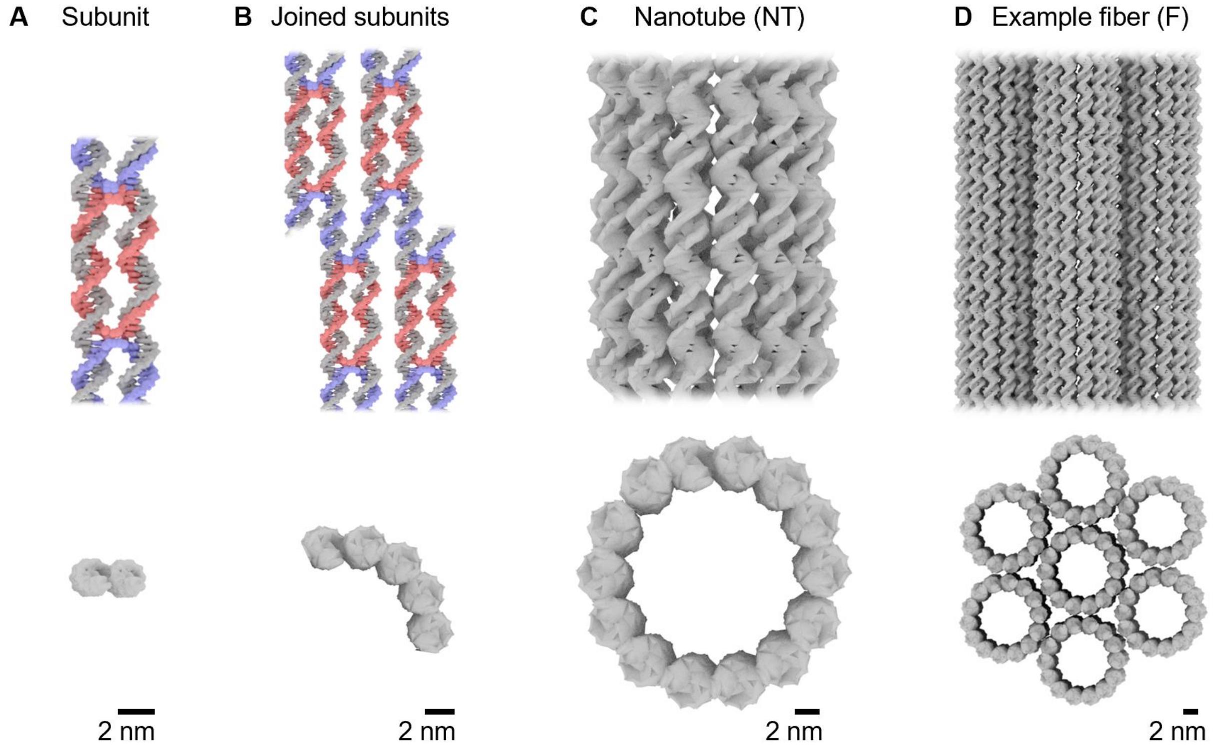

Figure S1. Rendering of higher order DNA structures with dimensions, side (top row) and profile (bottom row) views of A) subunit, B) four joined subunits, C) assembled 14 helical bundle nanotube and D) example fiber. All scale bars 2 nm. DNA nanotubes vary in diameter from 7-22 nm, for clarity the nanotube is represented as a 14 helix bundle.

1.5 2D map of subunit and joined subunits

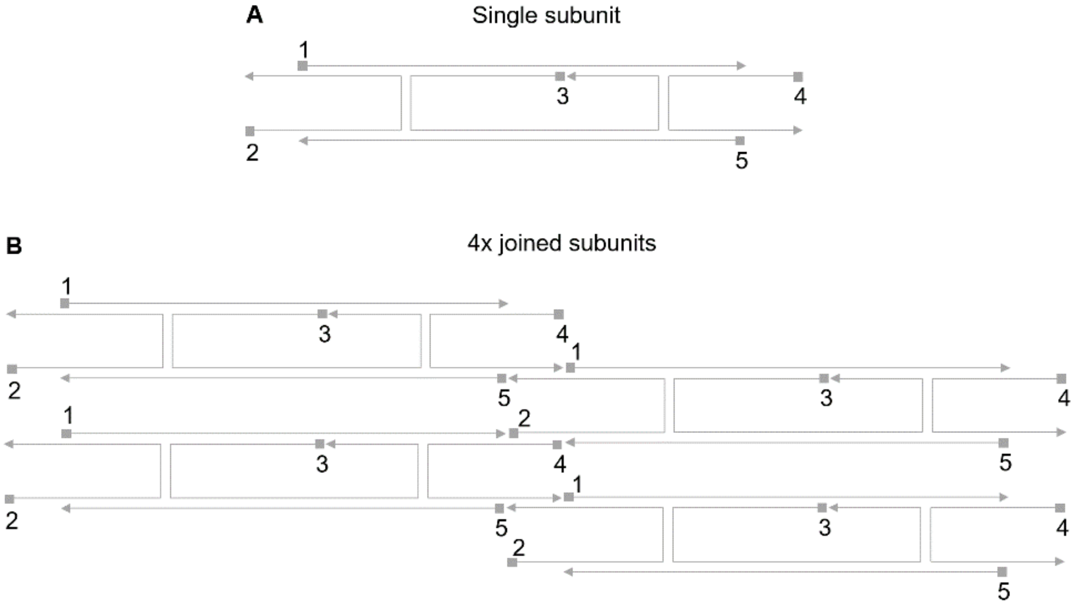

Figure S2. 2D maps of DNA nanotubes and fibers. A) Single subunit (gray) and B) 4x subunits joined *via* toe-hold extensions, strands are numbered 1 to 5, where the 5' and 3' termini are represented as squares and triangles, respectively.

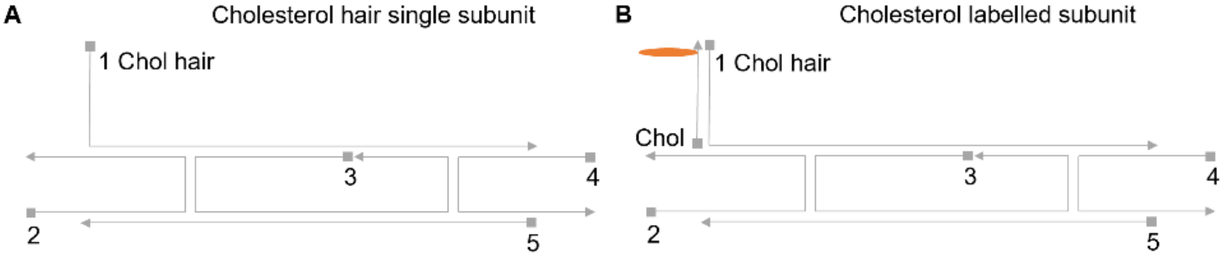

Figure S3. 2D maps of A) “hairy” subunit and B) cholesterol modified subunit, the core strands are numbered 1 Chol hair, and 2 to 5. A cholesterol PEG modified oligonucleotide (orange) was incorporated into the 3' terminus of strand Chol which formed a duplex with the single strand extended region of 1 Chol hair. The 5' and 3' termini are represented as squares and triangles, respectively.

2 Supplementary data

2.1 Structural characterization of DNA nanotubes and fibers

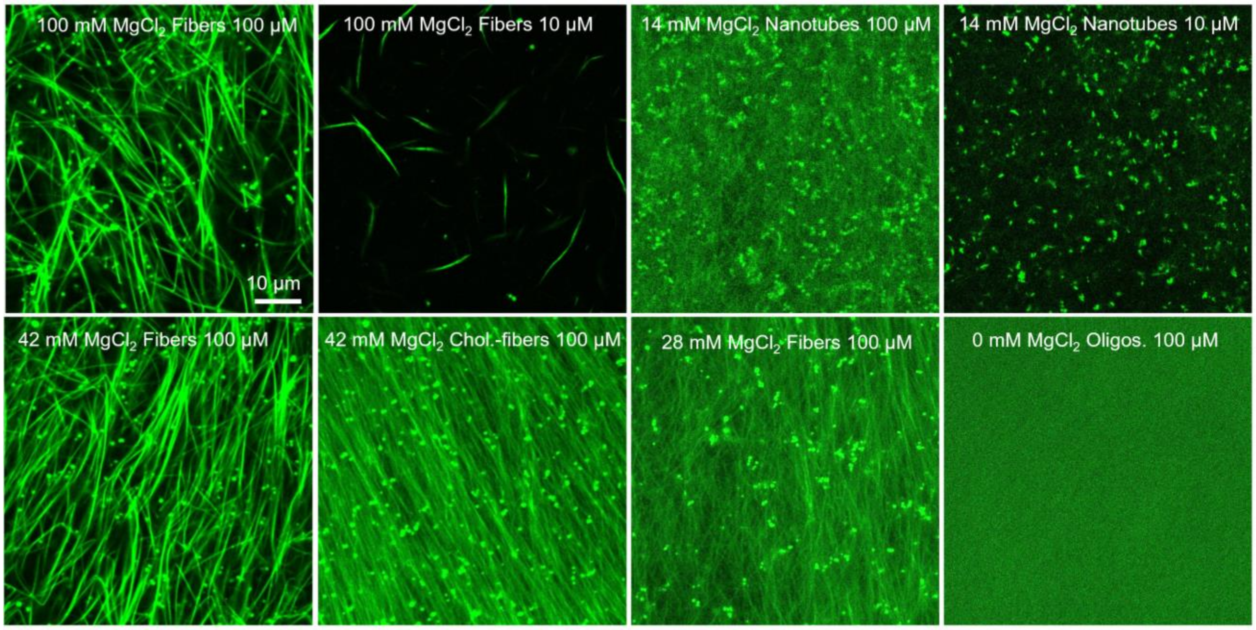

Figure S4. CLSM images of the stated Cy3-labeled DNA macrostructures at 100 µM or 10 µM where stated. Under these conditions, distinguishable fibers were observed at $MgCl_2$ concentrations of 28 mM and higher, nanotube bundles at 14 mM $MgCl_2$, and no observed DNA structures were identified at 0 mM $MgCl_2$. Scale bar 10 µm. The fibers can align on the surface of the dish due to lamellar flow resulting from the deposition process, this effect was exploited previously in a separate manuscript.[1] In addition, the DNA macrostructures can align above the surface of the dish due to their high concentration. A similar effect has been reported for protein cytoskeleton analogues.[2]

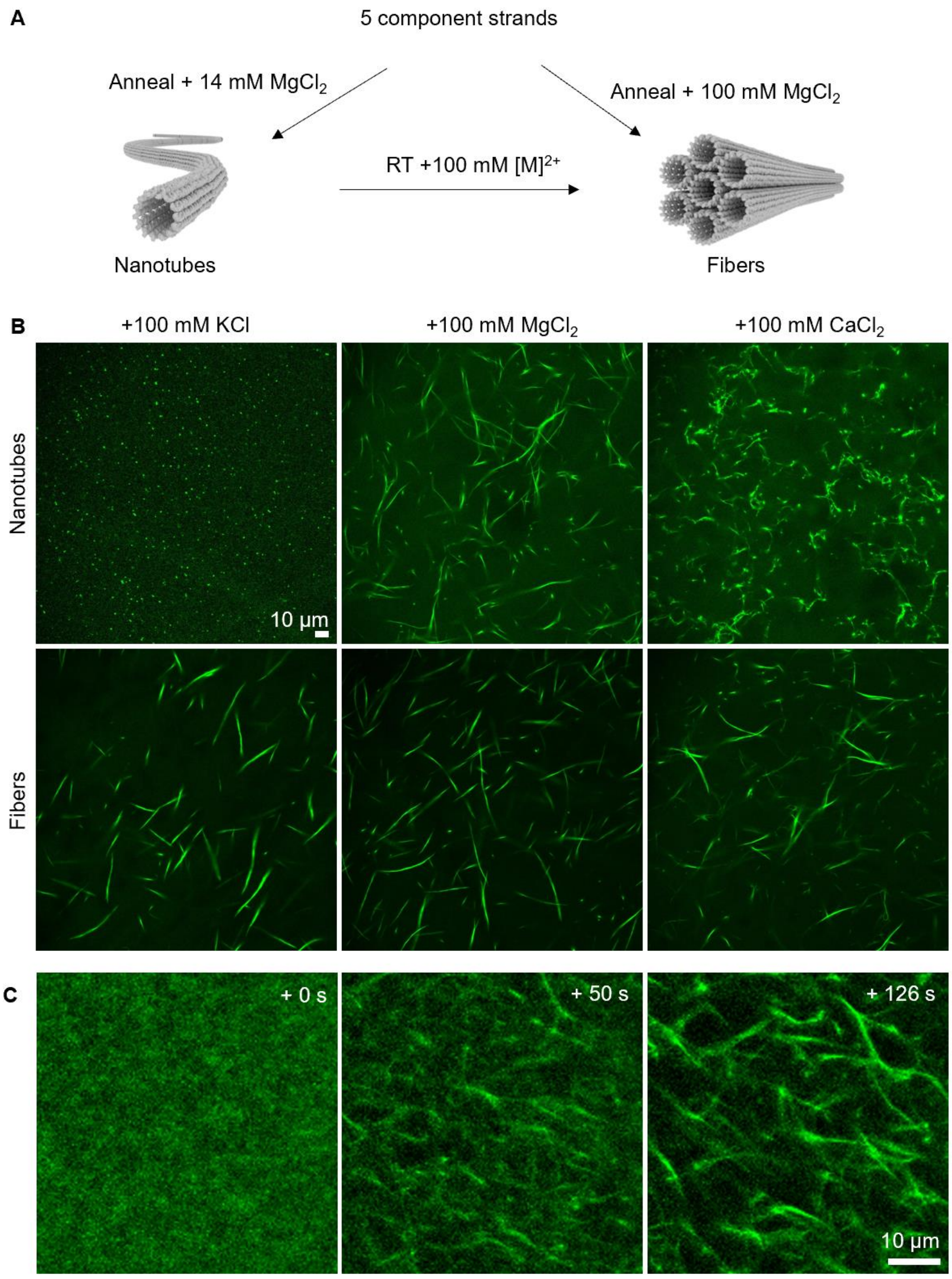

Figure S5. DNA nanotubes can be condensed into fibers after folding by adding divalent metals. A) Schematic reprepsentation, B) CLSM images showing either folded Cy3-labeled DNA nanotubes (top row) or fibers (bottom row), then mixed with the stated metal chloride at 100 mM, and C) CLSM time series of Cy3-labeled DNA nanotubes (5 µL, 25 µM) being condensed into fibers by adding magnesium chloride (5 µL of 200 mM $MgCl_2$) at room temperature, after 50 seconds distinguishable fibers were observed. All scale bars 10 µm.

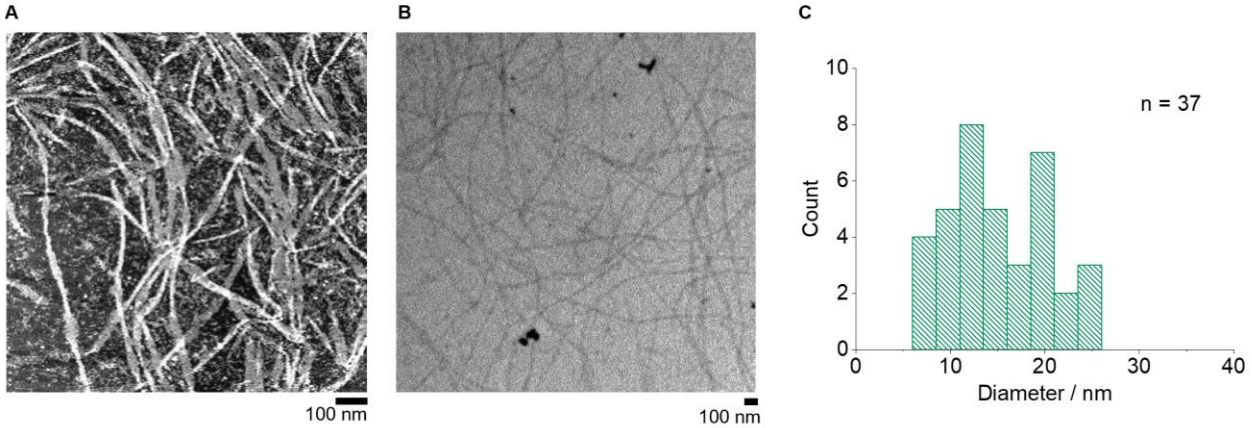

Figure S6. Structural characterization of 14 mM $MgCl_2$ DNA nanotubes. A) Atomic force microscope (AFM) image of DNA nanotubes at 1000 nM, scale bar 100 nm, under these conditions on the mica surface, nanotubes were able rupture and form a single layer lattice. B) Uranyl formate stained transmission electron microscope (TEM) image of DNA nanotubes at 1000 nM, scale bar 100 nm, and C) TEM-derived nanotube diameter histogram profile obtained from 37 measurements.

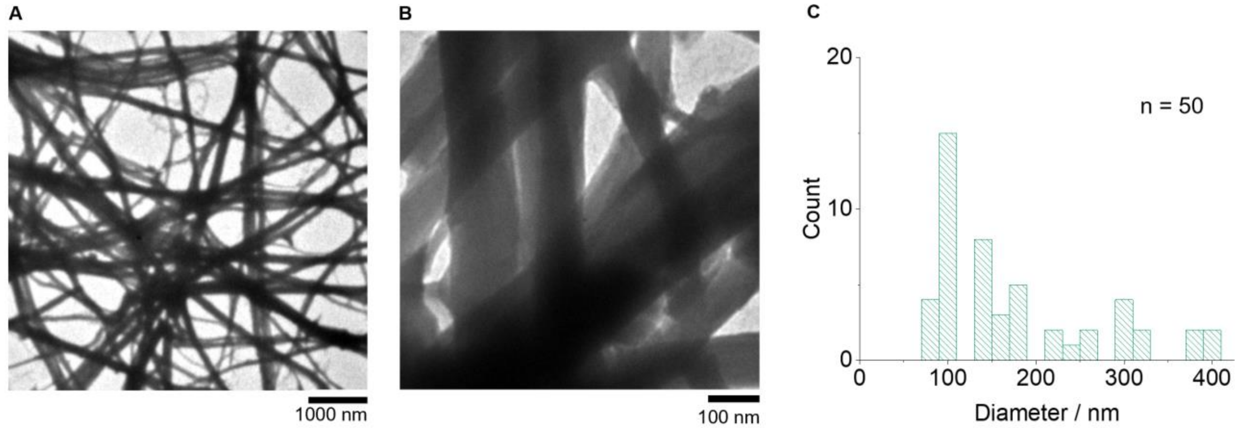

Figure S7. Structural characterization of 42 mM $MgCl_2$ DNA fibers. A) Uranyl formate stained TEM image of 42 mM MgCl2 DNA fibers at 1000 nM, scale bar 1000 nm, B) magnification, scale bar 100 nm, and C) corresponding fiber diameter histogram profile obtained from 50 measurements.

2.2 Solution-phase properties and formation yields of DNA macrostructures

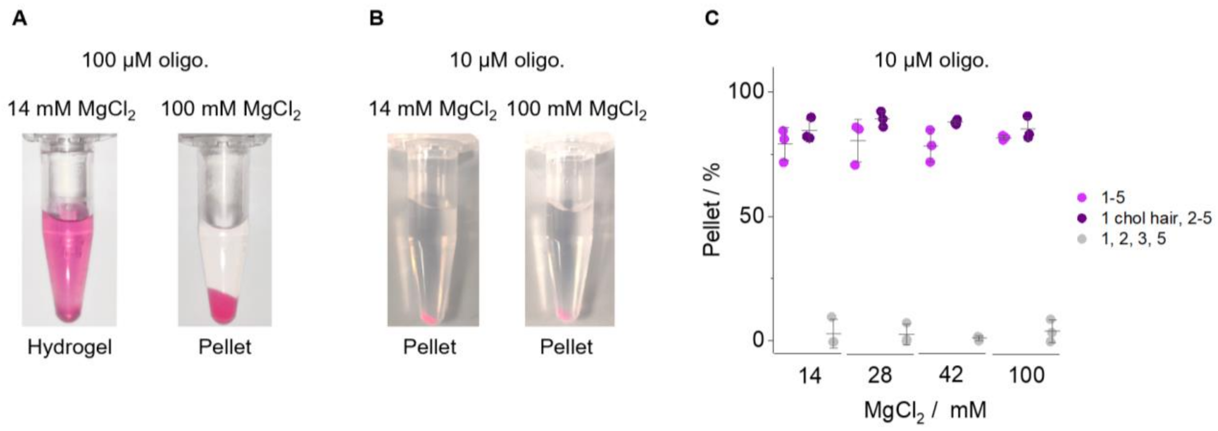

Figure S8. Investigating the pelleting properties of DNA macrostructures at high and low concentrations. Photographs of Cy3-labeled DNA nanotubes and fibers at A) 100 µM or B) 10 µM (component oligonucleotide concentration) after centrifugation in 1x TAE 14 mM $MgCl_2$ for nanotubes and 1x TAE 100 mM $MgCl_2$ for fibers, the nanotubes formed a hydrogel at 100 µM which did not pellet after centrifugaiton, but were soluble at 10 µM and did pellet after centrifugation. C) Percentage of the stated DNA macrostructure in the stated buffer and salt type large enough to pellet after 1x centrifugation pull-down, obtained by scaning the absorbance at 260 nm, for either non-hairy (1-5), hairy (1 chol hair, 2-5) and partial component strands (1, 2, 3, 5). Lines and error bars represent the median and standard deviation of 3 independent repeats.

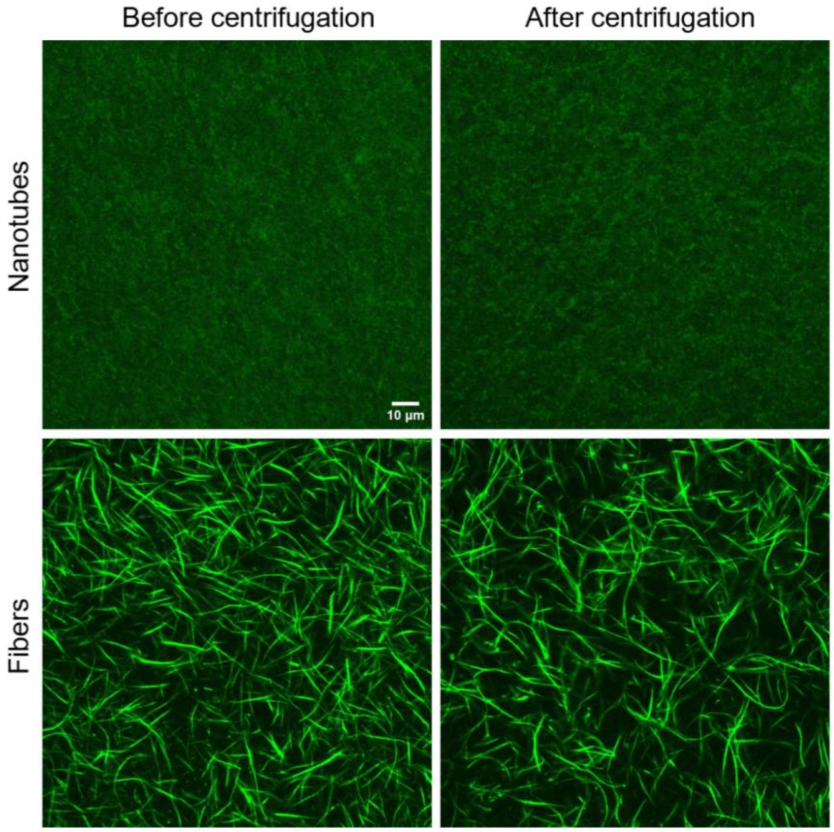

Figure S9. Centrifugation does not affect nanotubes (NT) or fibers (F) under the assayed conditions. CLSM images of the stated Cy3-labeled DNA macrostructures at 100 µM before and after 3x centrifugation-resuspension cycles. Scale bar 10 µm.

2.3 CLSM analysis of protocells

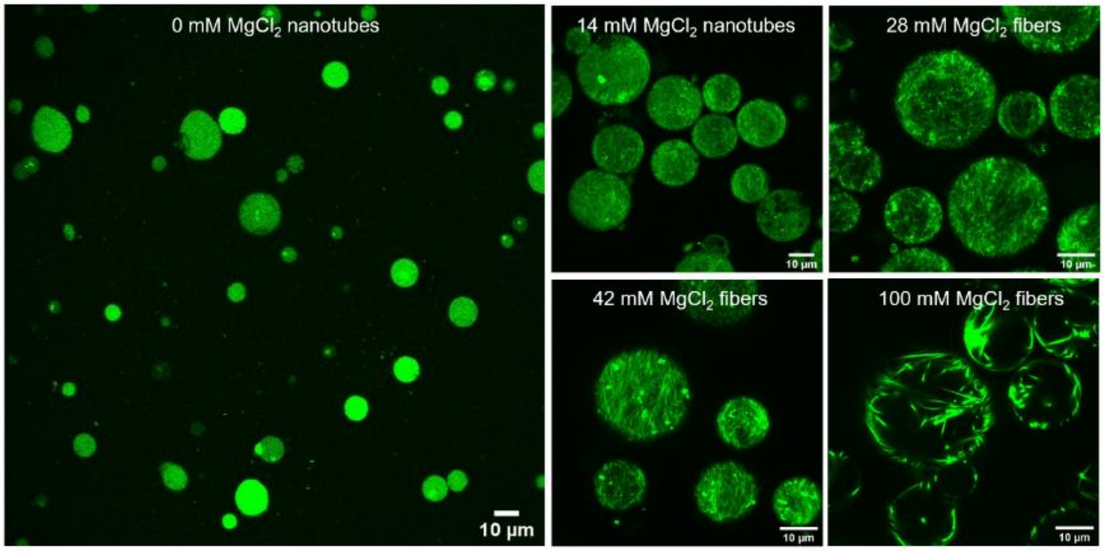

Figure S10. CLSM images of the stated Cy3 labeled-DNA cytoskeleton structures inside POPG protocells generated using the inverted emulsion approach. Distinguishable DNA macrostructure textures were observed at 14 mM $MgCl_2$ and above, but not for the control, 0 mM $MgCl_2$. All scale bars 10 µm.

2.4 Melting profiles of 42 mM $MgCl_2$ DNA fibers

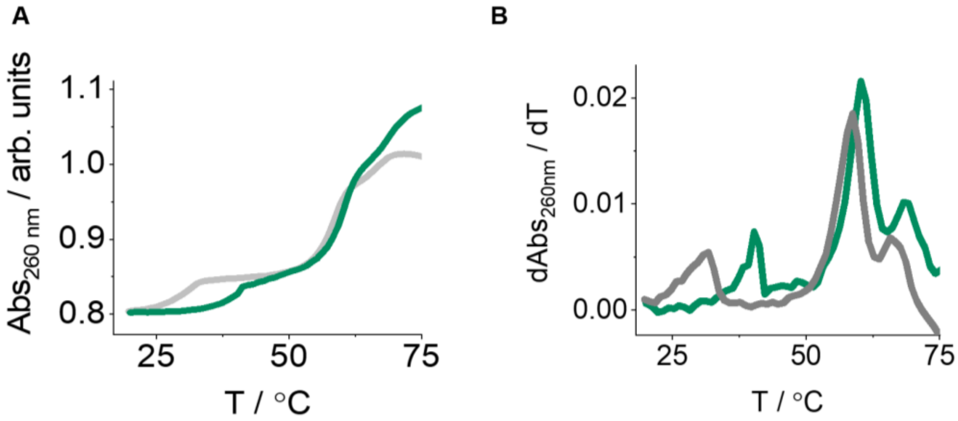

Figure S11. A) 260 nm UV absorbance melting (green line) and annealing (gray line) profiles, and B) corresponding first derivatives of 42 mM $MgCl_2$ DNA fibers. Sample was diluted to 1 µM in 50 % v/v 725 mM sorbitol and 50 % v/v 42 mM $MgCl_2$ TAE pH 8.3. The two annealing transitions observed at 40 and 60°C correspond to the fiber polymerization and subunit formation steps, respectively.[1]

2.5 Assembling DNA fibers inside protocells

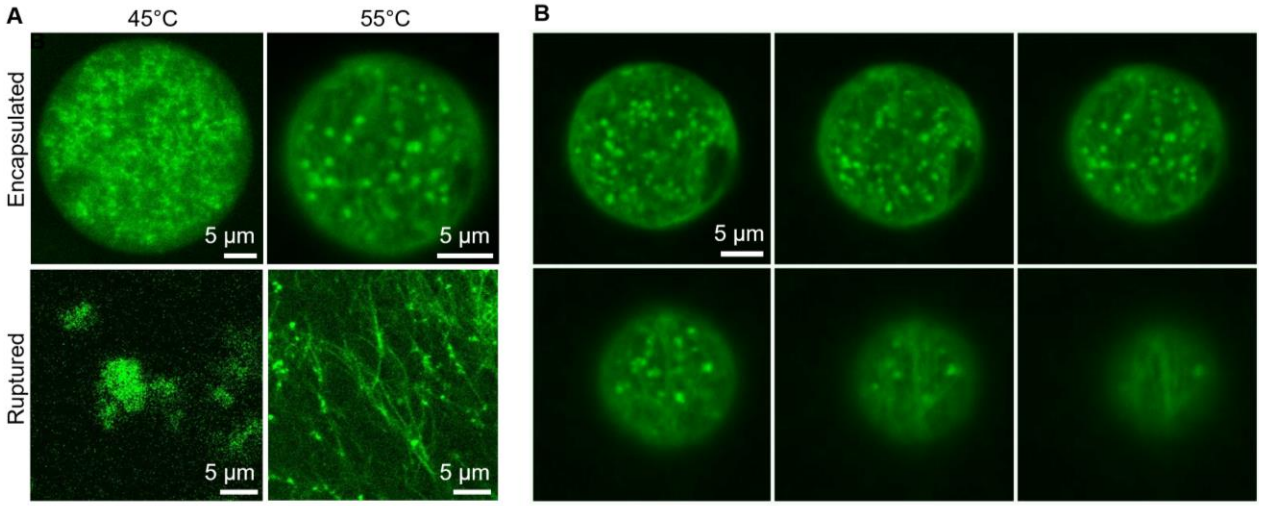

Figure S12. A) CLSM images showing compartmentalized DNA fibers can be assembled inside GUVs (top row) by annealing from 55°C (>T_M), but not from 45°C (<T_M), and corresponding DNA structures after rupturing with 0.1 % v/v Triton-X100 (bottom row). B) Z-stack montage taken from A after heating to 55°C showing fibers textures. Scale bars 5 µm.

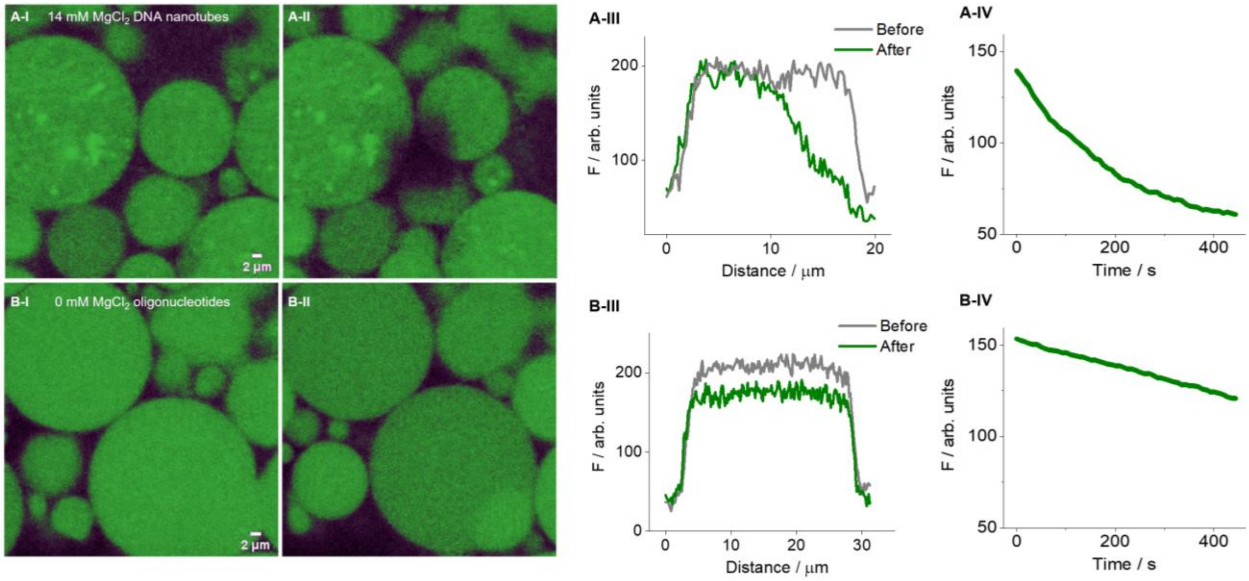

Figure S13. FLIP analysis of nanotube and oligonucleotide protocells. CLSM-derived fluorescence loss in photobleaching (FLIP) of compartmentalized DNA structures for A) 14 mM $MgCl_2$ DNA nanotubes and B) 0 mM $MgCl_2$ oligonucleotides, A-I) and B-I) CLSM images before FLIP and A-II) and B-II) CLSM images after FLIP, A-III) and B-III) corresponding line section

profiles of each bleached region, and A-IV) and B-IV) FLIP bleaching region of interest fluorescence intensity profiles overtime. Only nanotubes displayed well-defined and localized bleached region. Scale bars 2 µm.

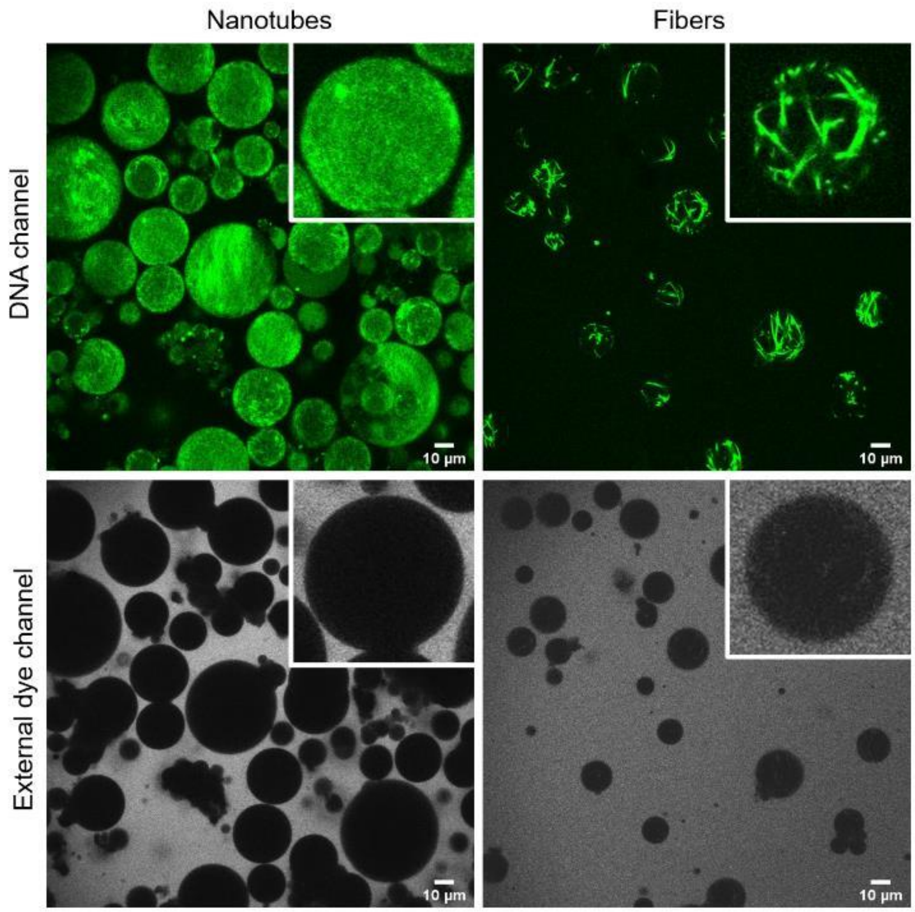

Figure S14. Confirming protocell membrane integrity using an external dye. CLSM images of stated POPG protocells containing either cytoskeletal 14 mM $MgCl_2$ DNA nanotubes (left column) or 100 mM $MgCl_2$ DNA fibers (right column) generated using the inverted emulsion approach, Cy3-labeled DNA cytoskeleton channel (top row, green) and small molecule dye, 5,6-carboxyfluorscein channel (bottom row, gray) added to the exterior to confirm protocell membrane tight seal was maintained. All scale bars 10 µm.

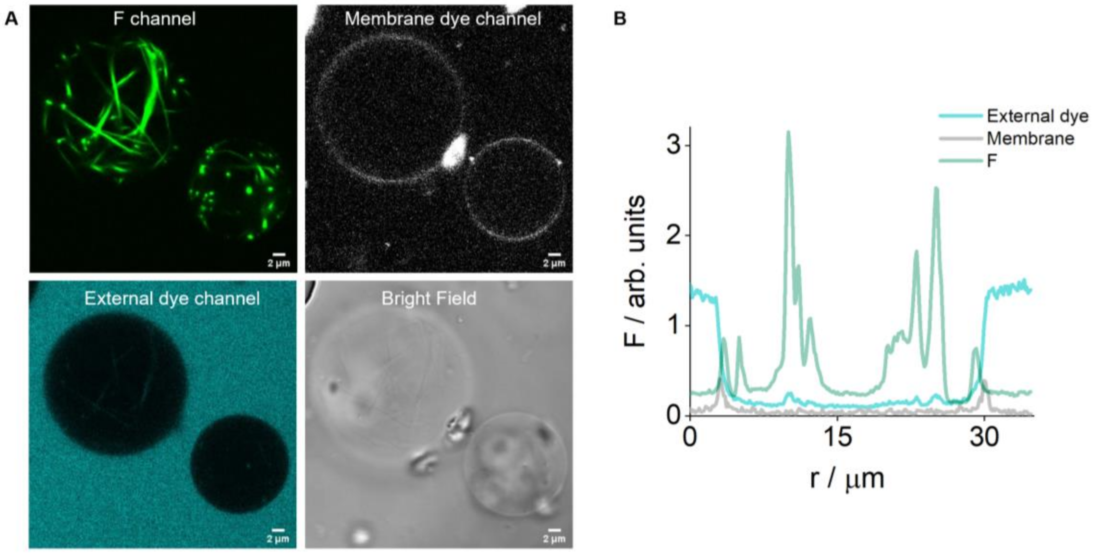

Figure S15. Confirming protocell membrane integrity using a membrane dye and external dye. A) CLSM images of Cy3-labeled 100 mM $MgCl_2$ DNA fibers (F, green channel) POPG GUVs with Cy5-PE lipid membrane dye (gray channel), external small molecule dye 5,6-carboxyfluorscein (cyan channel) and bright field image, and B) corresponding line section profiles of distance (r) plotted against fluorescence from the stated channels in A. Images confirmed membrane tight seal maintained throughout. All scale bars 2 µm.

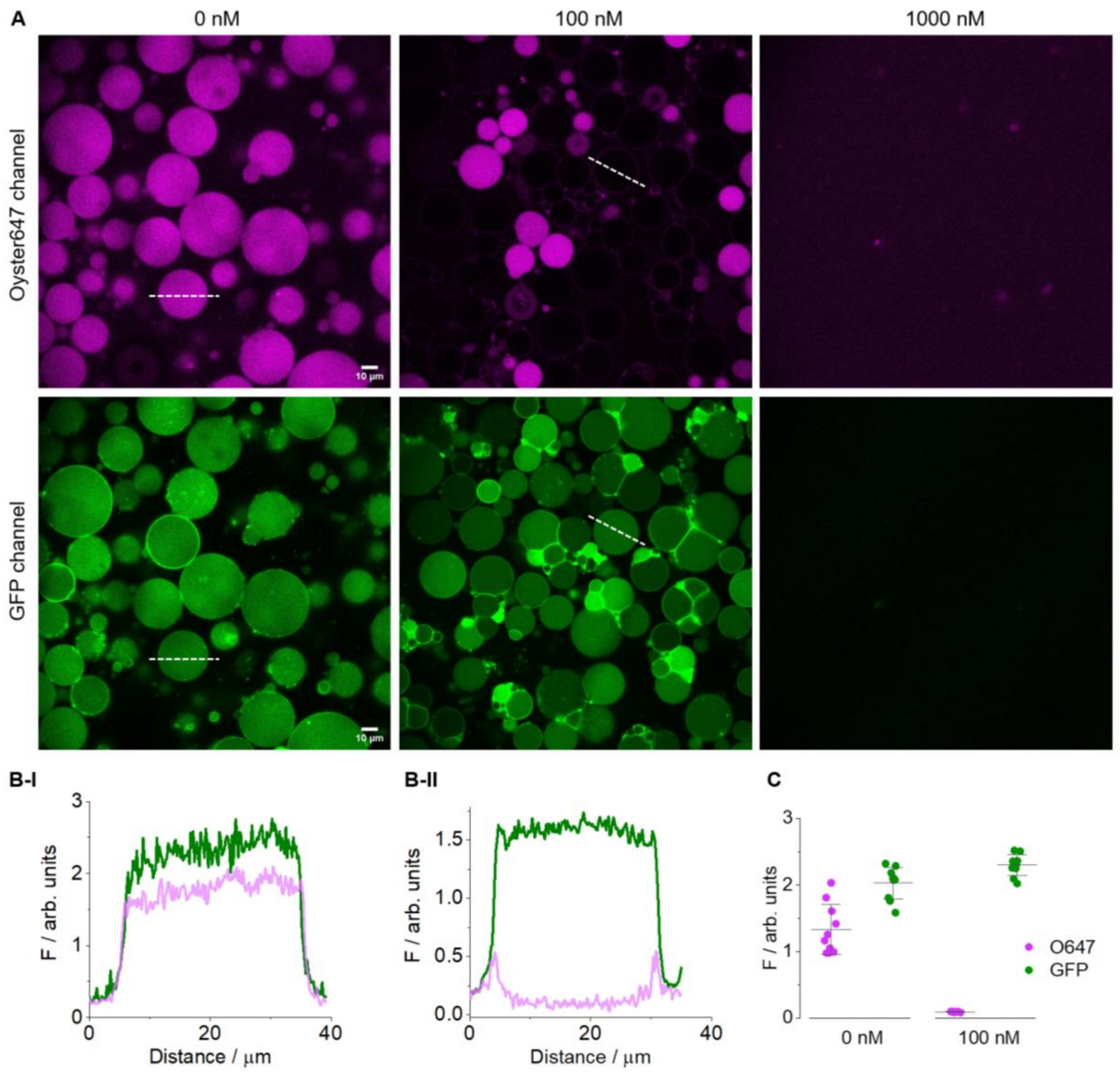

Figure S16. Confirming the unilamellar nature of protocell membranes using a protein nanopore. A) CLSM images of cytoskeletal 14 mM $MgCl_2$ DNA nanotube protocells co-encapsulated with Oyster647 (O647) (top row, magenta channel) and GFP (bottom row, green channel) at either 0, 100 or 1000 nM alpha-hemolysin (αHL), incubated for 60 min at room temperature. B) Corresponding line section profiles of selected protocells for O647 (magenta) and GFP (green) at B-I) 0 nM and B-II) 100 nM αHL. C) Dot plot showing fluorescence intensities inside protocells at 0 or 100 nM αHL for GFP (green) and O647 (magenta). Line and error bars represent median and standard deviation of 11 measurements which showed transport, respectively. All scale bars 10 µm.

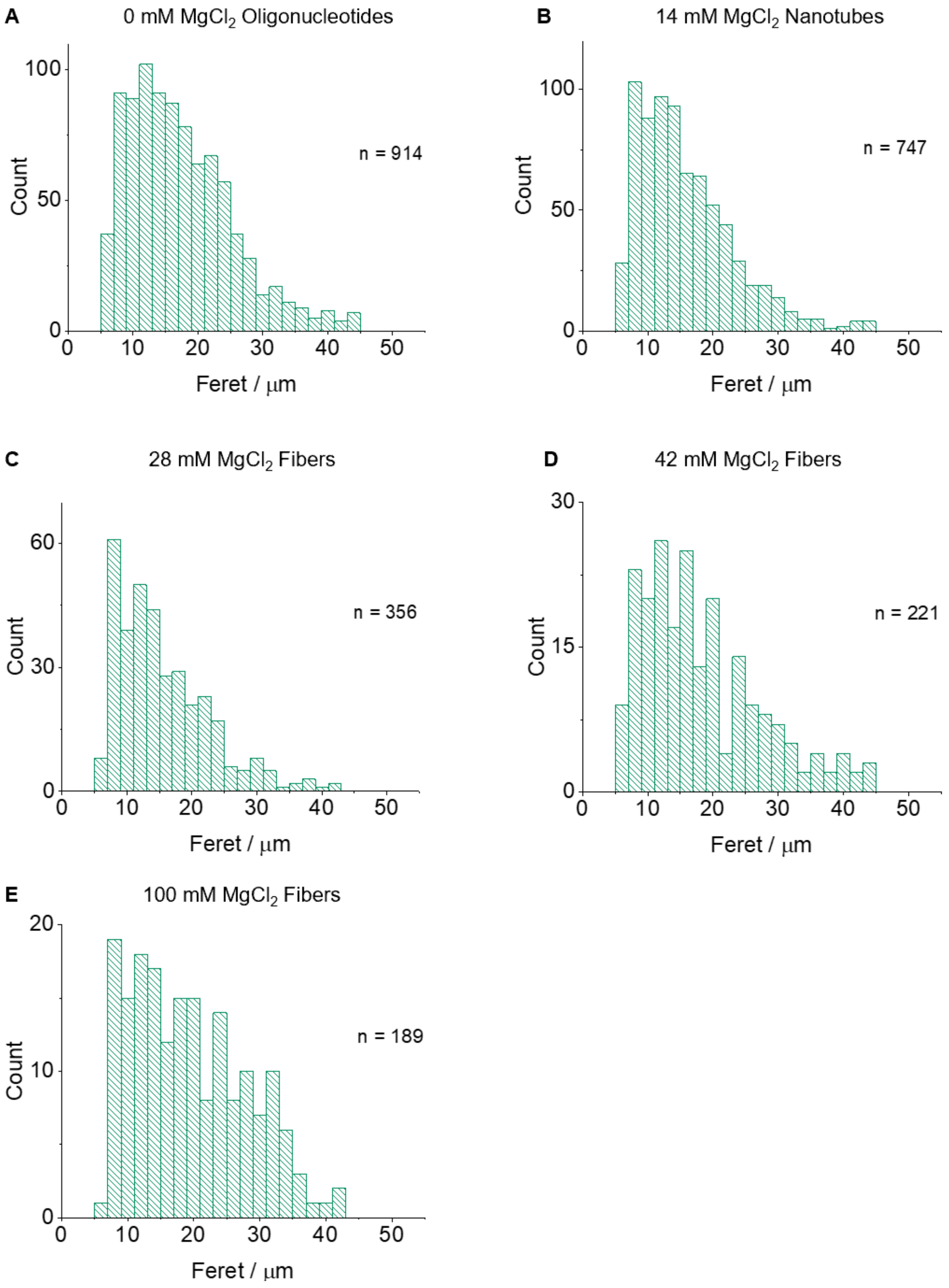

Figure S17. CLSM-derivied Feret diameter histogram plots of DMPC : cholesterol : POPG protocells containing compartmentalized A) 0 mM $MgCl_2$ oligonucleotides, B) 14 mM $MgCl_2$ nanotubes, C) 28 mM $MgCl_2$ fibers, D) 42 mM $MgCl_2$ fibers and E) 100 mM $MgCl_2$ fibers. n value represents total number of counts in each plot.

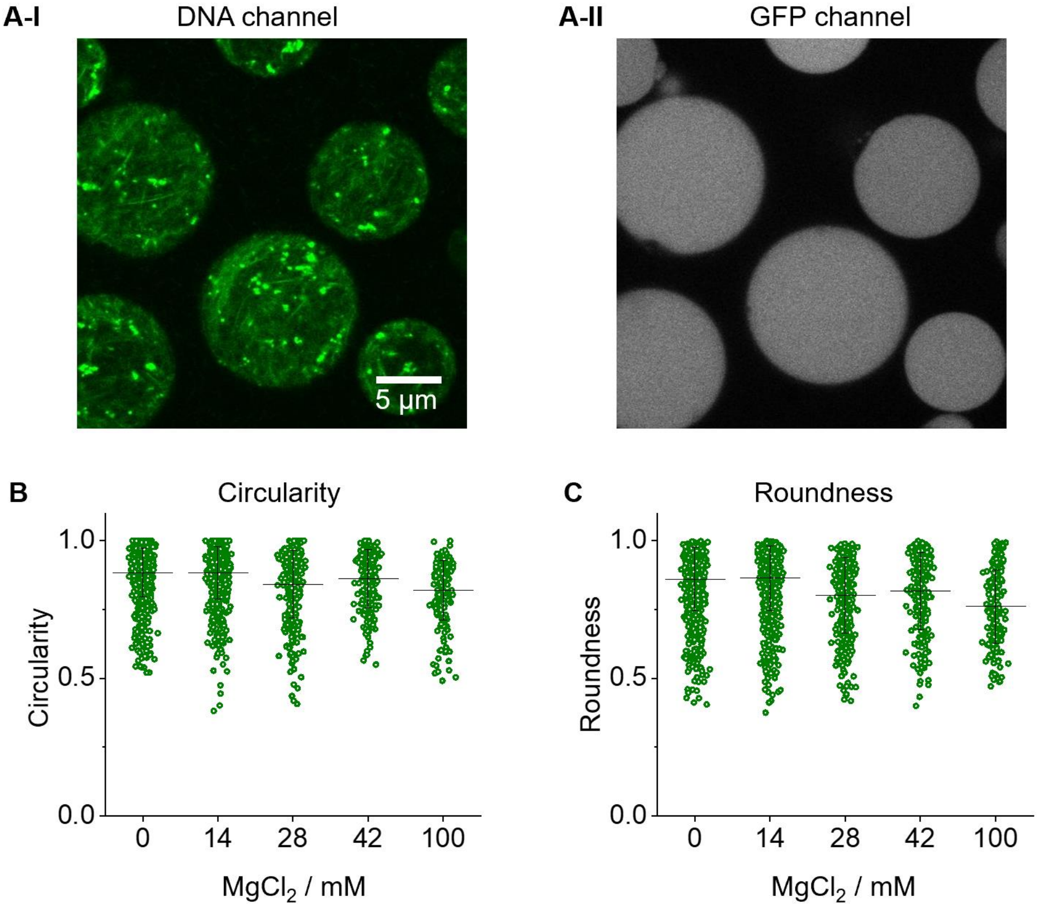

Figure S18. CLSM-derived roundess and circulairty analysis of protocells usng GFP co-encapsulated protocells. A) Example CLSM images of A-I) Cy3-labeled 28 mM $MgCl_2$ DNA fibers channel and A-II) GFP channel used to quantify protocell properties, corresponding B) circularity and C) roundness dot plots for protocells containing either 14 mM $MgCl_2$ DNA nanotubes, 28 mM $MgCl_2$ fibers, 42 mM $MgCl_2$ fibers and 100 mM $MgCl_2$ fibers plotted in Figure S17, line and error bars represent the median and standard deviation, repectively. Scale bar 5 µm.

2.6 Determining protocell lipid generalizability

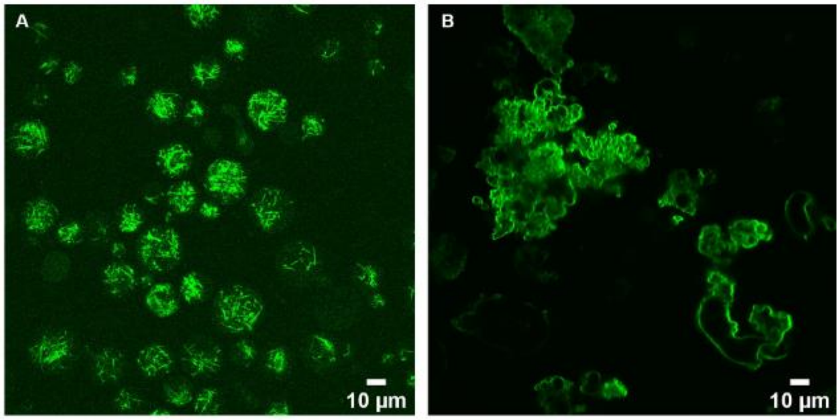

Figure S19. CLSM images confirmed cytoskeleton Cy3-labeled 100 mM $MgCl_2$ DNA fibers are compatible with DMPC cholesterol (2 : 1 molar ratio) when fibers were transferred prior to encapsulation into A) 1.4 mM $MgCl_2$ 1x TAE pH 8.3, B) but collapased to form non-spherical vesicles in 14 mM $MgCl_2$ 1x TAE pH 8.3 or higher. Scale bars 10 µm.

2.7 Determining protocell osmolar stability and rupturing properties

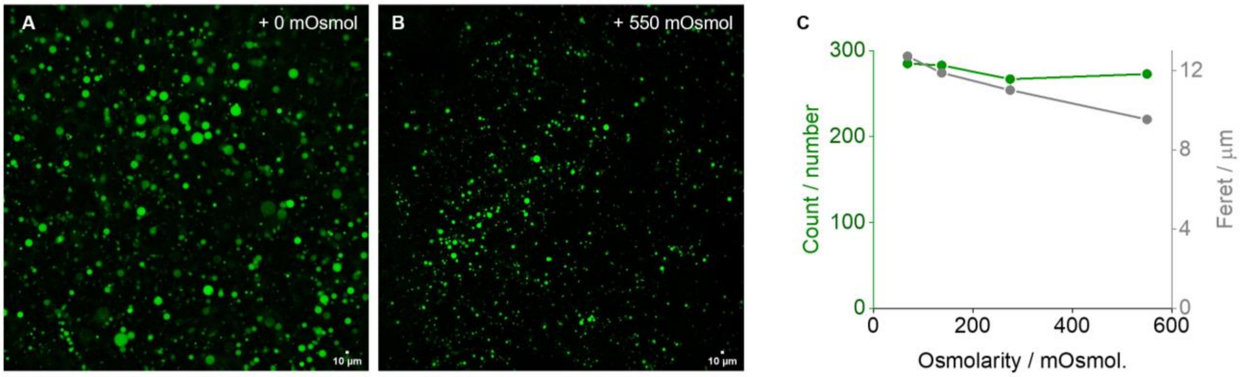

Figure S20. Ultra-hyperosmolarity assay to determine cytoskeleton stability. Representative CLSM images of cytoskeleton 14 mM $MgCl_2$ DNA nanotube DMPC : cholesterol : POPG protocells containing internal GFP dye (green channel), at either A) + 0 mOsmol or B) + 550 mOsmol, and C) corresponding number of protocells remaining (green axis) and Feret diameter (gray axis) derived at the stated mOsmol. Scale bars 10 µm.

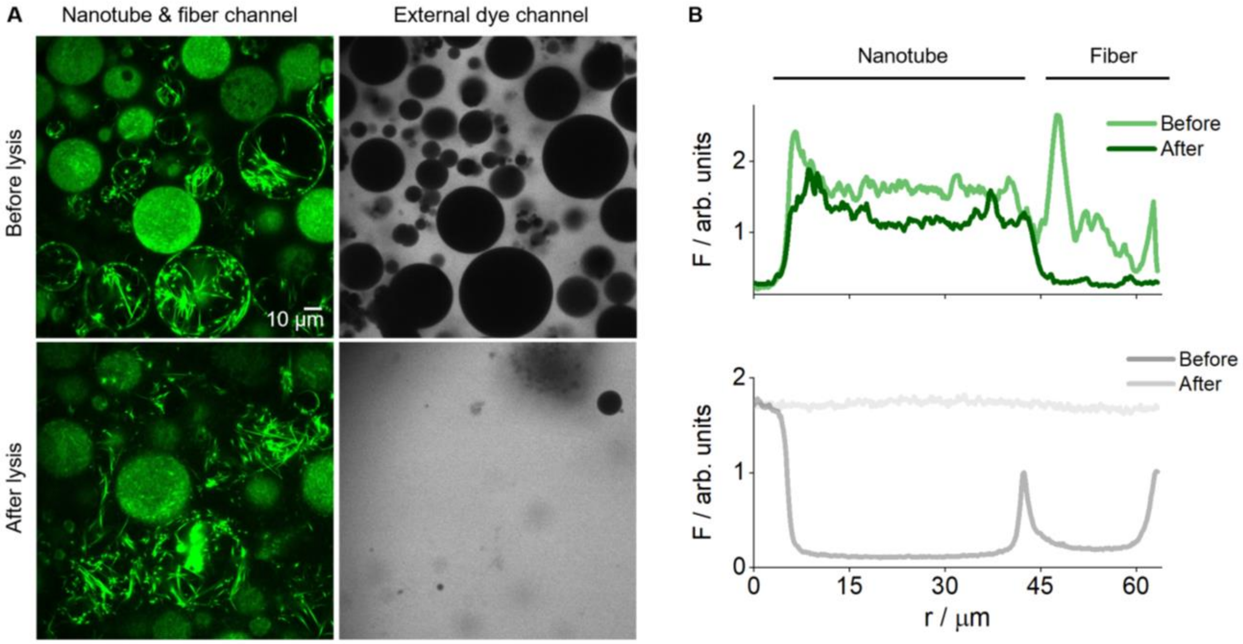

Figure S21. Exploring protocell lysis. A) CLSM images of Cy3 labeled cytoskeleton nanotube or fiber (green channel, left column) protocells with external dye (gray channel, right column), before (top row) and after addition of lysis agent, Triton-X100 (bottom row), and B) corresponding line section profiles derived from A for the stated channels. After protocell lysis only nanotube cytoskeletons retained their spherical shape. Scale bar 10 µm.

2.8 Generating multi-DNA cytoskeleton protocells

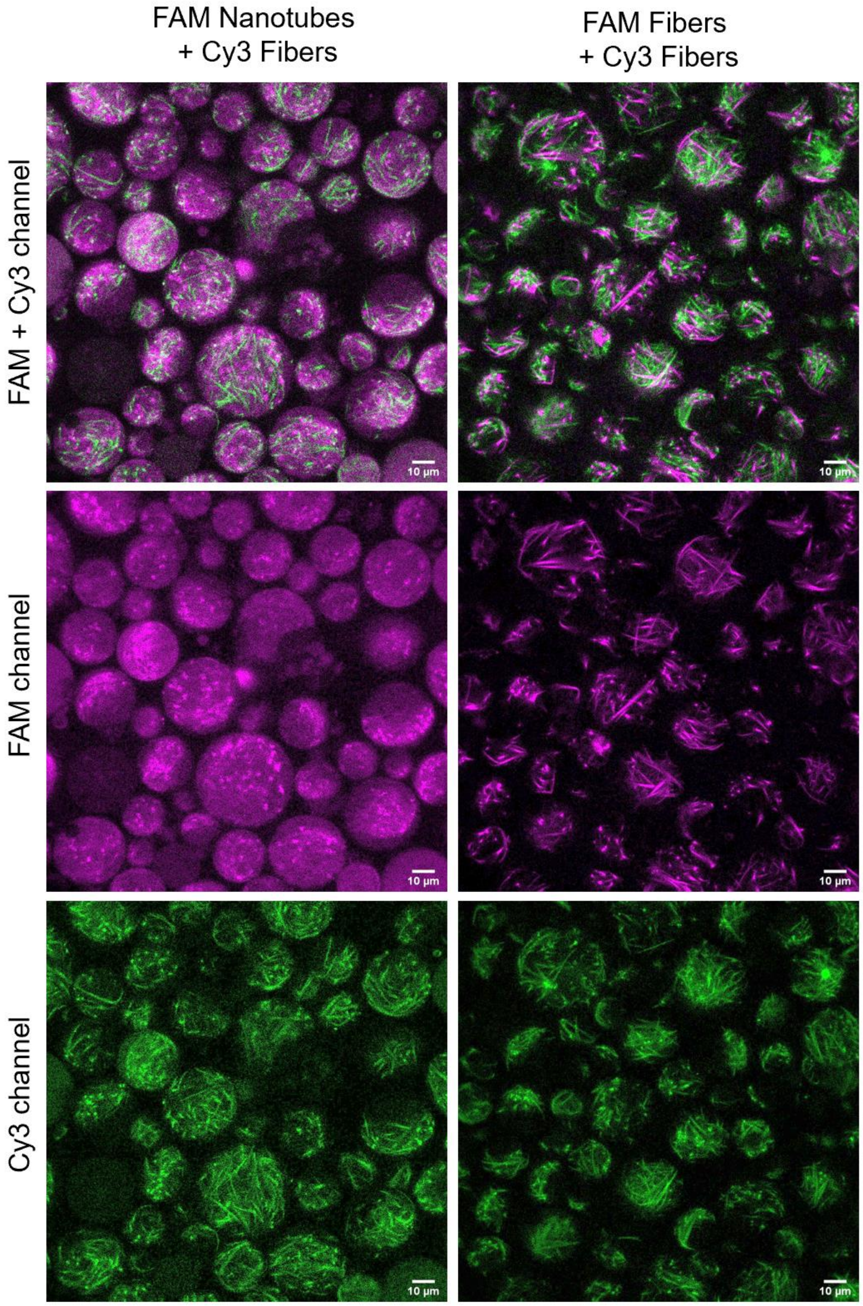

Figure S22. Multiple DNA macrostructures can be compartmentalized inside the same protocell. CLSM analysis of compartmentalized FAM-labeled 14 mM $MgCl_2$ DNA nanotubes (magenta channel) and Cy3-labeled 100 mM $MgCl_2$ fibers (green channel) (left column), and two types (FAM and Cy3 labeled) of 100 mM $MgCl_2$ DNA fibers encapsulated inside DMPC : cholesterol : POPG protocells (right column). Scale bars 10 µm.

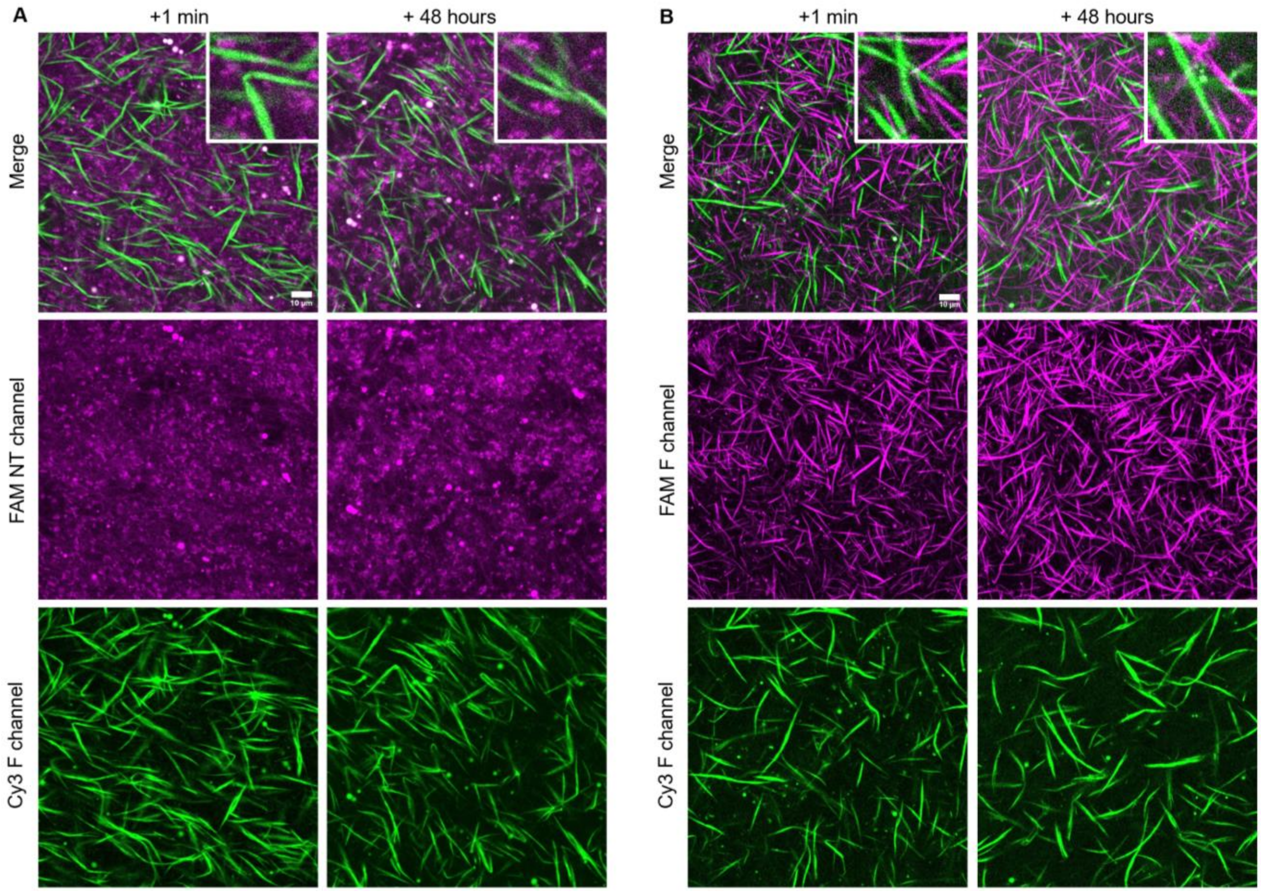

Figure S23. DNA nanotubes and fibers are not in equilibrium after folding under assayed conditions. CLSM images of A) FAM-labeled 14 mM $MgCl_2$ nanotubes (magenta channel) and Cy3-labeled 100 mM $MgCl_2$ fibers (green channel), or B) FAM-labeled 100 mM $MgCl_2$ fibers and Cy3-labeled 100 mM $MgCl_2$ fibers, insets show 3.5x magnification. All constructs were mixed in a 1 : 1 molar ratio at 50 µM for either 1 min or 48 hours and then imaged. Scale bar 10 µm.

2.9 Compartmentalized proximity control of cholesterol DNA fibers

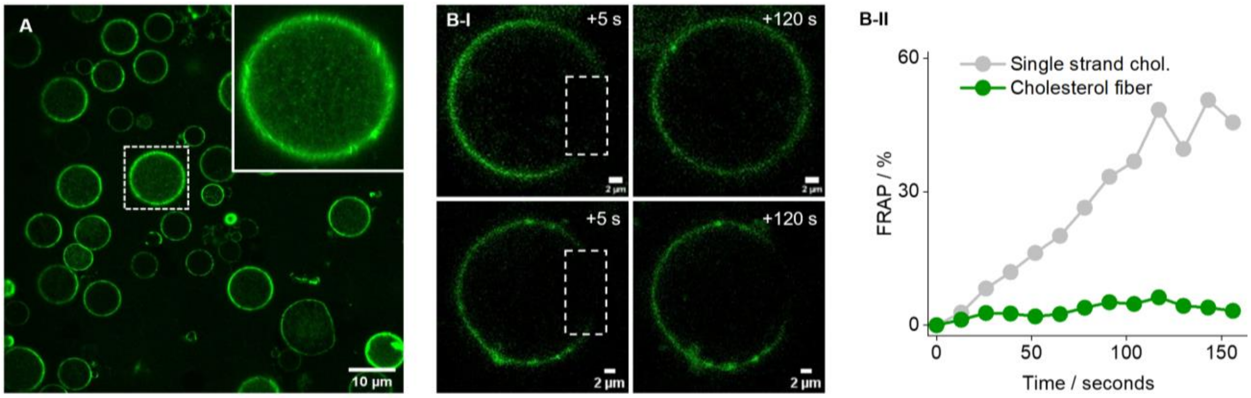

Figure S24. A) CLSM images of FAM-labeled 42 mM $MgCl_2$ cholesterol DNA fibers (green channel) compartmentalized inside DMPC : cholesterol : POPG protocells and magnified region of interest (inset) which showed defined fiber textures along the membrane periphery, scale bar 10 µm. B-I) FRAP images of either FAM-labeled single strand cholesterol (top row) and 42 mM $MgCl_2$ cholesterol DNA fibers (bottom row), 5 (left column) or 120 seconds (right column) after the laser-induced photobleaching event, dotted boxes denotes the bleached region of interest, all scale bars 2 µm. B-II) Corresponding FRAP profiles obtained from B-I, single strand cholesterol (gray line) and 42 mM $MgCl_2$ cholesterol fibers (green line), only single strand cholesterol, but not cholesterol fibers, displayed a fluorescence recovery profile within this timeframe.

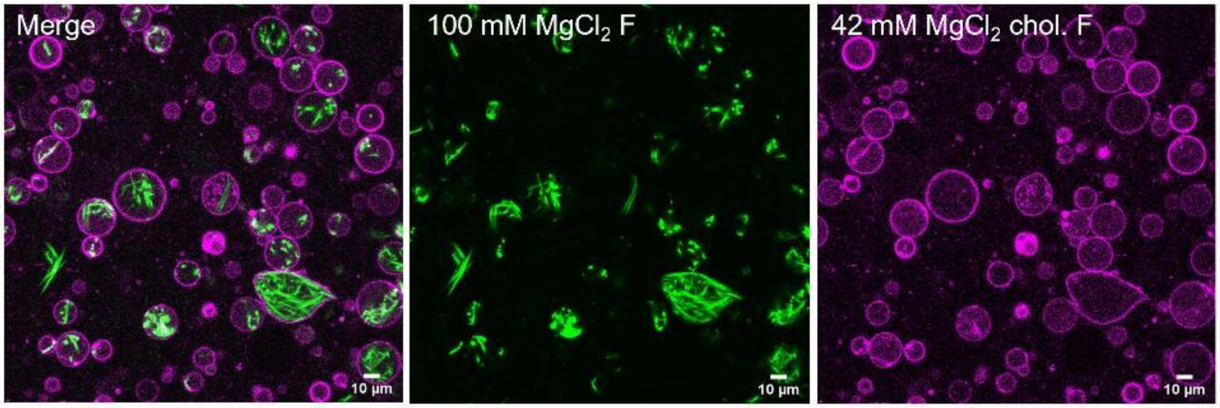

Figure S25. CLSM images of FAM-labeled 42 mM $MgCl_2$ cholesterol DNA fibers (magenta channel) and Cy3 labeled 100 mM $MgCl_2$ DNA fibers (green channel) co-compartmentalized inside DMPC : cholesterol : POPG protocells. Only cholesterol labeled DNA macrostructures exhibited membrane colocalization. All scale bars 10 µm.

2.10 Magnetic particle coated fibers inside protocells

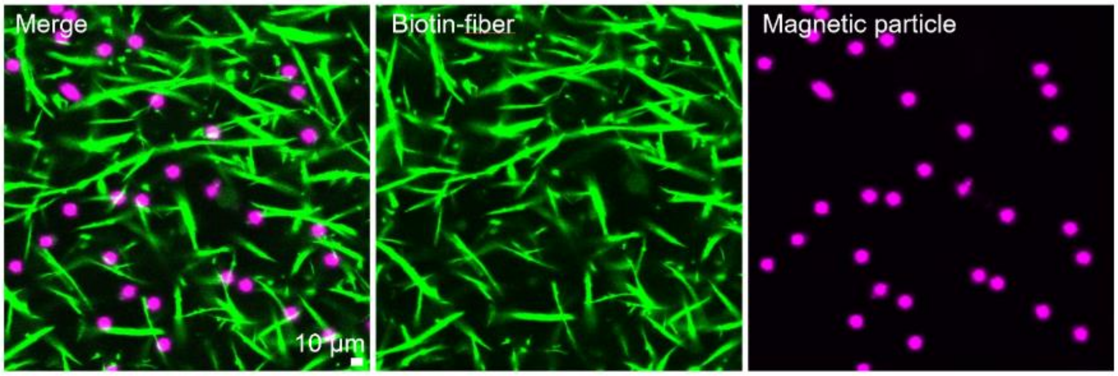

Figure S26. CLSM images of biotin and Cy3-labeled 100 mM $MgCl_2$ DNA fibers (green channel) with magnetic particles (magenta channel). Images show the DNA fibers are not affected by the biotin modifications, and the magnetic particles colocalize specifically along DNA fibers. Scale bar 10 µm.

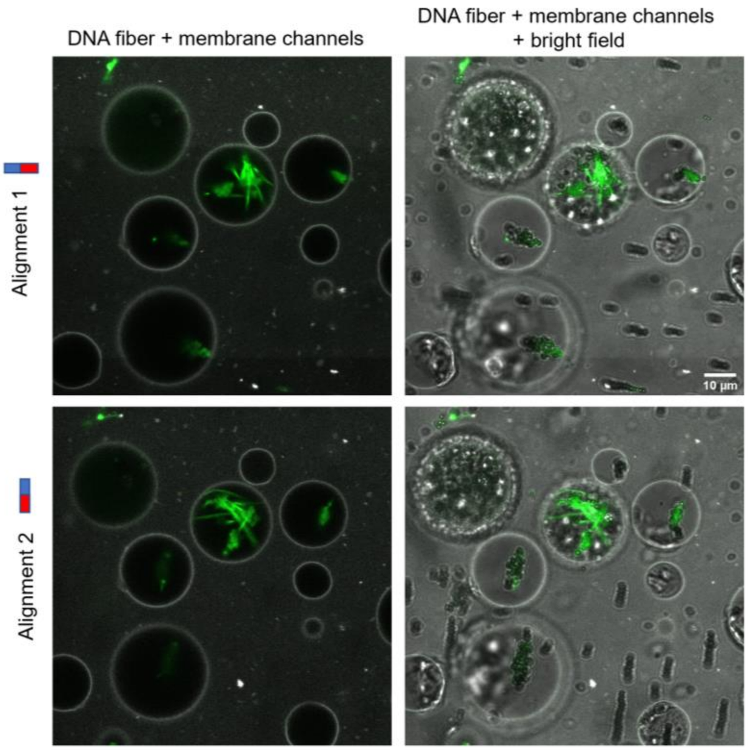

Figure S27. Controlling DNA fiber alignment using magnetic particles with an external magnetic field. CLSM images of Cy3 labeled magnetic particle biotin 100 mM $MgCl_2$ DNA fibers (green channel) inside POPG GUVs containing Cy5 PE lipid (gray channel), with either east-west (top row) or north-south (bottom row) alignment with an external magnetic bar. Bright field image shows the alignment of the magnetic particles. Scale bar 10 µm.

2.11 Vesicle-in-vesicle cytoskeleton supported protocells

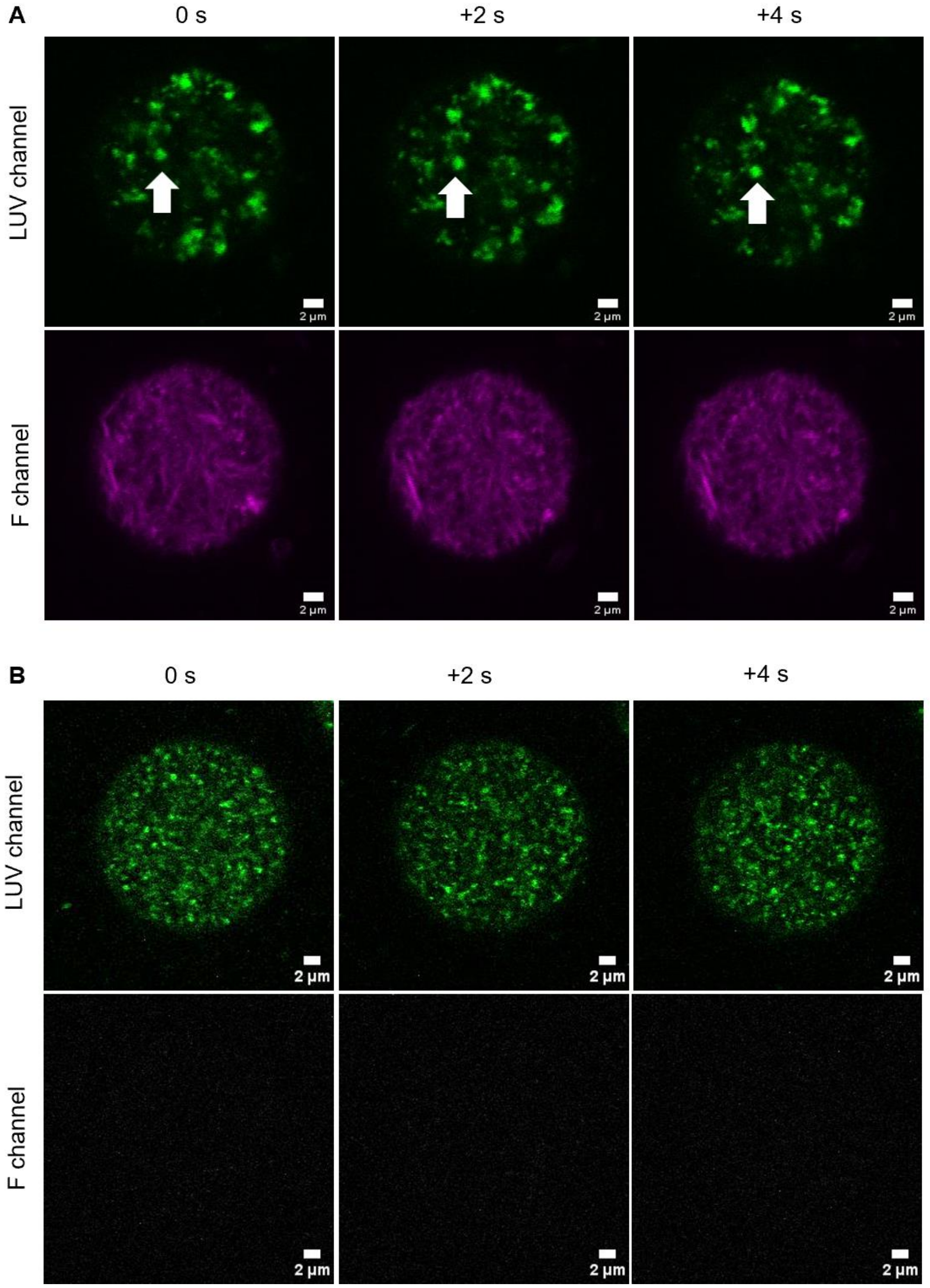

Figure S28. Cytoskeleton fibers bind and support internal LUVs within protocells. CLSM time series of DMPC : cholesterol : POPG protocells with internalized 2 µm diameter extruded LUVs containing FAM lipid dye (green channel) either A) with or B) without Cy3-labeled 100 mM DNA fibers (magenta channel). LUVs adhered to DNA fibers between frames (see arrow), but rapidly move inside protocells without a cytoskeleton support. Scale bar 2 µm.

2.12 DNA fiber exoskeleton GUVs

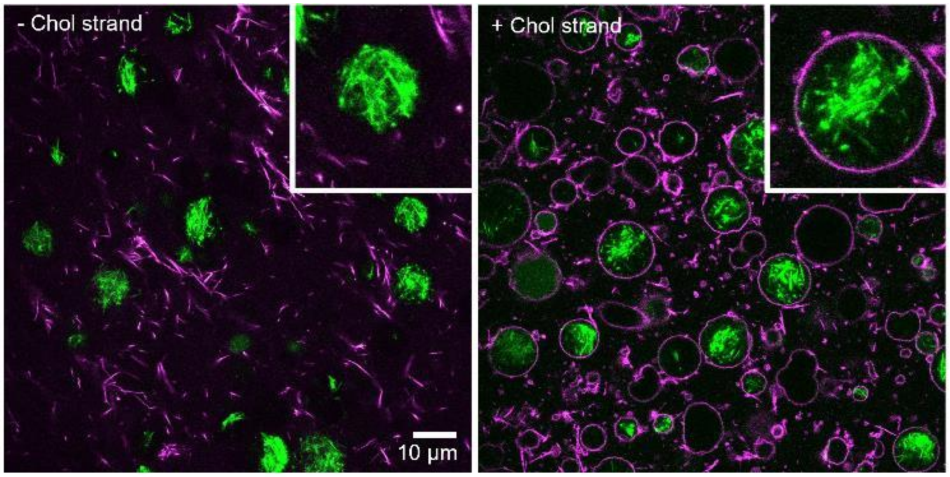

Figure S29. Controlling exoskeleton-fiber membrane colocalisation with cholesterol lipid anchor DNA. CLSM analysis of FAM labeled exoskeleton DNA fibers (magenta channel) and Cy3-labeled cytoskeleton 100 mM $MgCl_2$ DNA fibers (green channel) inside POPG protocells, either without (left), or with (right) cholesterol lipid anchor DNA strand. Scale bar 10 µm.

2.13 Determining nuclease resistant properties of exoskeleton protocells

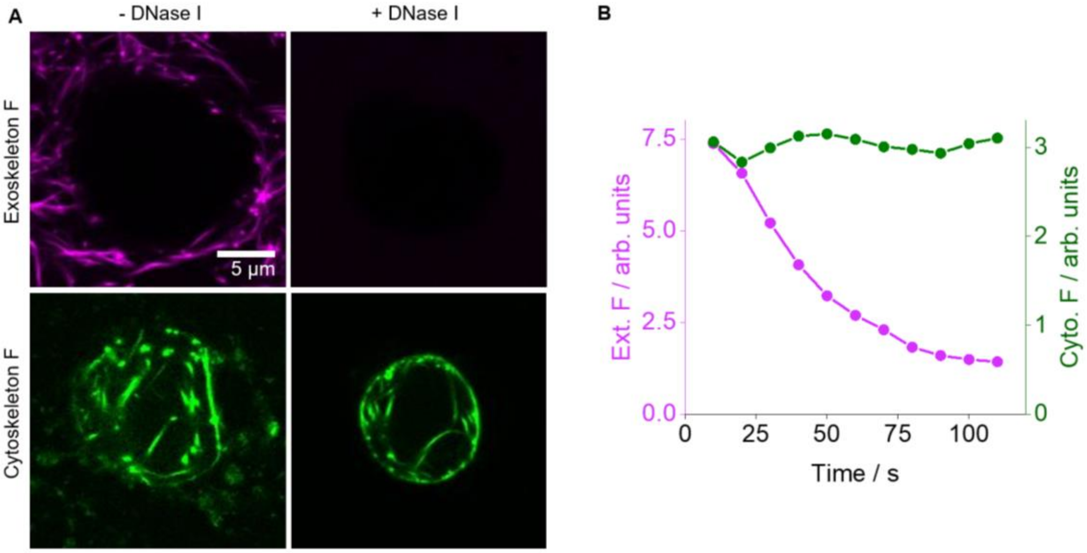

Figure S30. Nuclease resistant properties of cytoskeleton DNA fibers. A) CLSM images of FAM labelled 100 mM DNA fiber exoskeletons (magenta channel) and Cy3 labeled 100 mM $MgCl_2$ DNA fibers cytoskeletons (green channel), before and after DNase I addition, and B) corresponding fluorescence intensity time series profiles after the addition of DNase I. Scale bar 5 µm.

2.14 Rupturing properties of dye-loaded vesicles

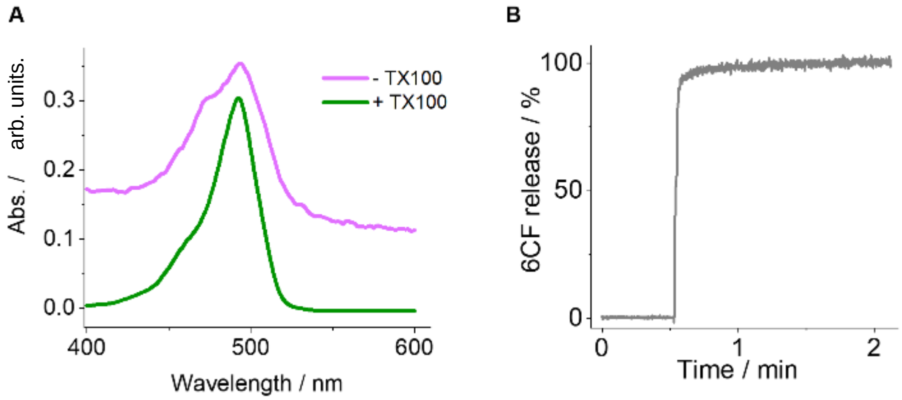

Figure S31. Rupturing properties of 200 mM 5,6-carboxyfluorescein encapsulated POPG vesicles. A) Absorbance profiles of vesicles before (magenta line) and after rupturing by adding 0.1 % v/v Triton-X100 (green line) and B) corresponding kinetic fluoresce emission plot, 0.1 % v/v Triton-X100 was added at 30 s giving rise to a rapid increase in emission upon vesicle rupturing.

2.15 DNA fibers in high glucose

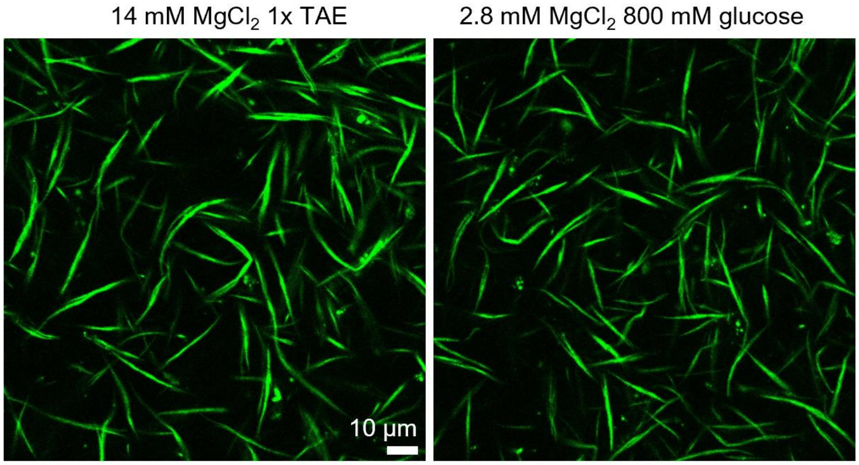

Figure S32. CLSM images of Cy3-labeled DNA fibers in the stated media. Images show DNA fibers are not affected by the extreme high glucose concentration experienced during the EIC process. Scale bar 10 µm.

2.16 Prototissue particle tracking analysis

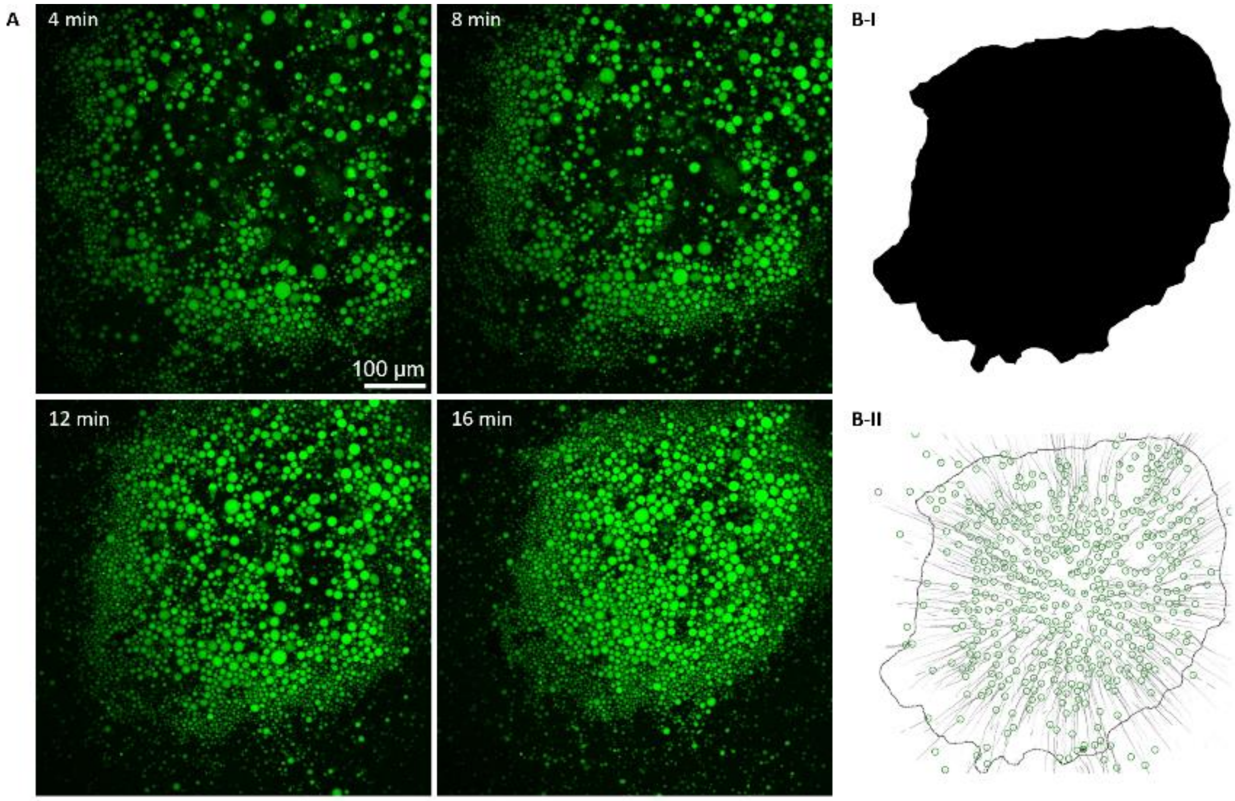

Figure S33. Monitoring prototissue formation in real time. A) CLSM time series showing images at selected time intervals, B-I) prototissue surface area obtained from A at 16 min extrapolated using ImageJ diameter analysis tool, and B-II) particle tracking map derived from time series showing final prototissue perimeter (black line), and constituent protocell final location (green circles) and their proceeding paths (gray lines), obtained using TrackMate software.[3] All scale bars 100 μm.

2.17 Controlling prototissue formation

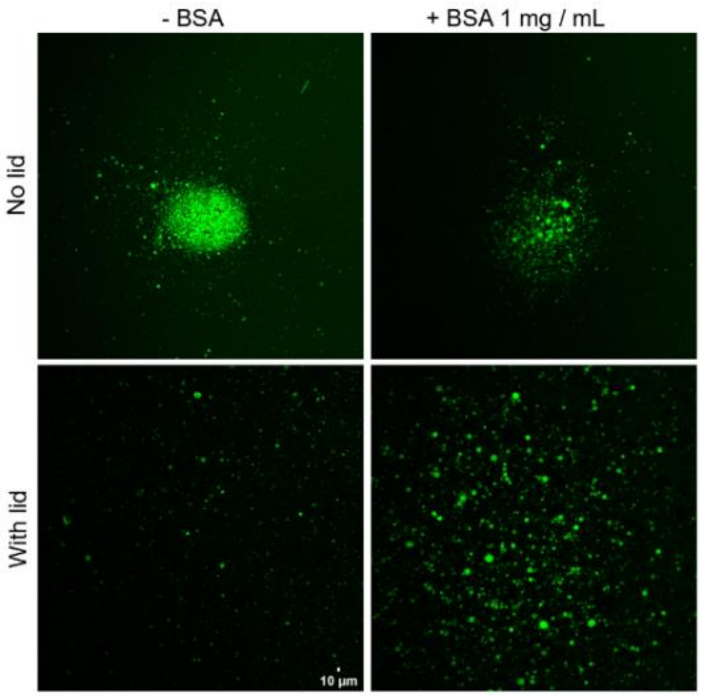

Figure S34. Determining mechanism of prototissue formation. CLSM images of protocells after 30 min at room temperature either with (top row) or without (bottom row) evaporation induced convection (EIC), and in the absence (left column) or presence (right column) of bovine serum albumin (BSA) at 1 mg per mL. Scale bar 10 µm. Only in the absence of BSA and without a lid did well defined prototissues form under these conditions.

2.18 Generating multi-layered prototissues

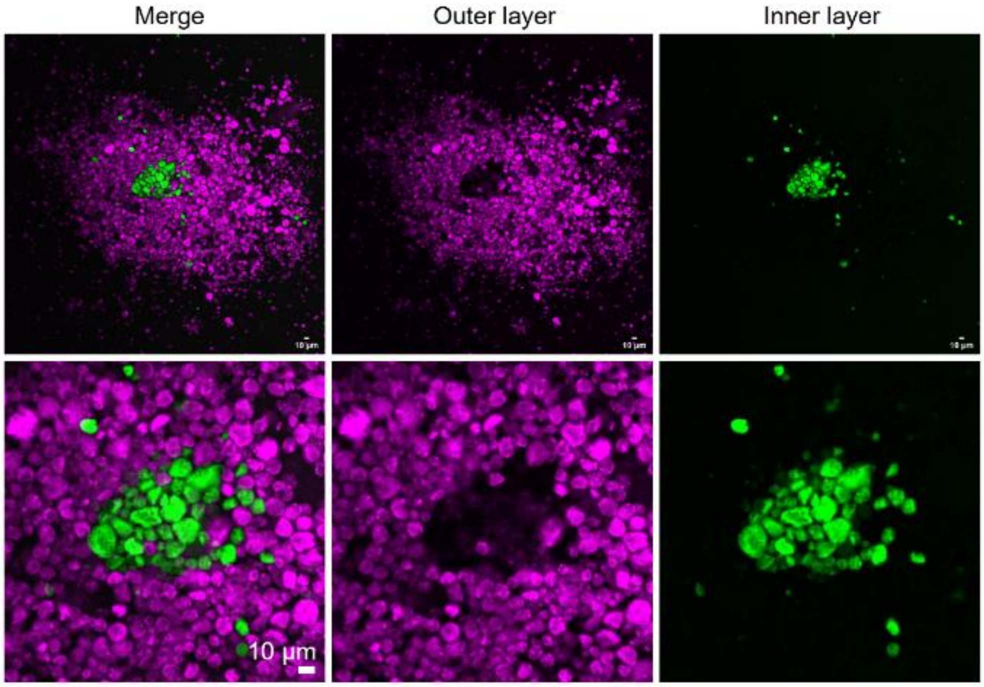

Figure S35. CLSM images of two types of DMPC : cholesterol protocells deposited 30 min apart which formed a multi-layered prototissue, the inner layer (green channel) contained internal GFP, and outer layer (magenta channel) contained cytoskeleton nanotubes only. All scale bars 10 µm.

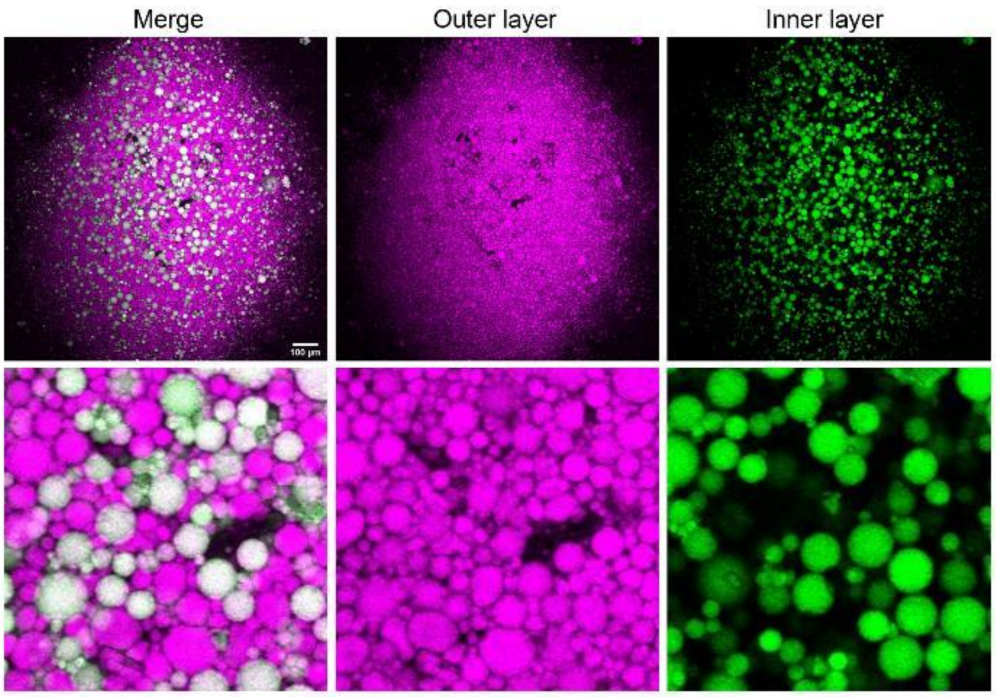

Figure S36. CLSM images of two types of DMPC : cholesterol protocells deposited at the same time which formed a heterogeneous prototissue. All scale bars 100 µm.

2.19 Building prototissues of different sizes

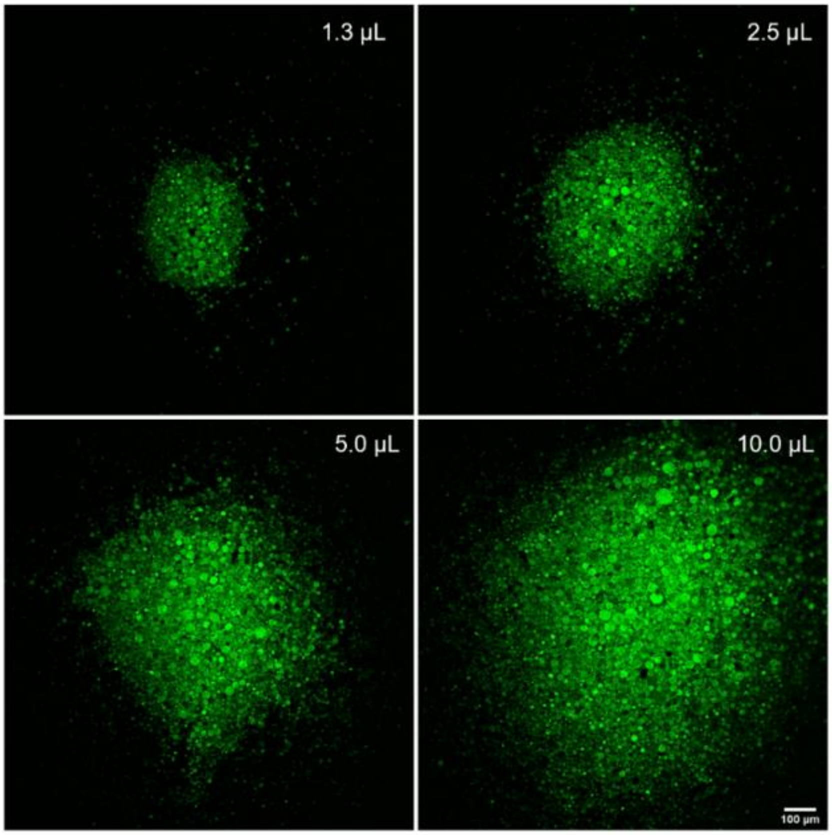

Figure S37. Prototissue dimensions correlate linearly to the amount of GUV deposited. CLSM images of prototissues (cytoskeleton green channel) containing the stated amounts of GUV supplemented with either 8.7, 7.5, 5 and 0 µL of 400 mM glucose, left to settle without the lid on for 30 min then imaged. Scale bar 100 µm.

2.20 Idenfitying the influence of exoskelelton fibers during prototissue formation

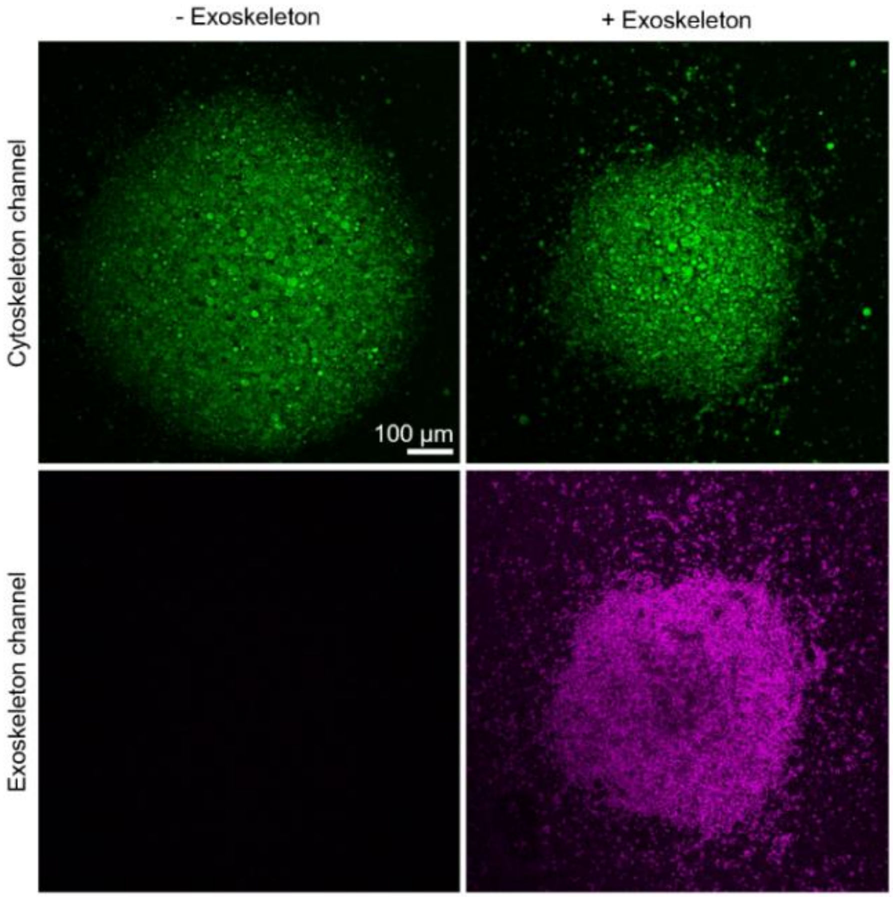

Figure S38. CLSM images monitoring the final prototissue size for protocells without (left column) or with (right column) exoskeletons, cytoskeleton (top row) and exoskeleton channels (bottom row). Image show the exoskeleton reduces the prototissue size. Scale bar 100 μm.

2.21 Investigating prototissue packing with different exoskeletons

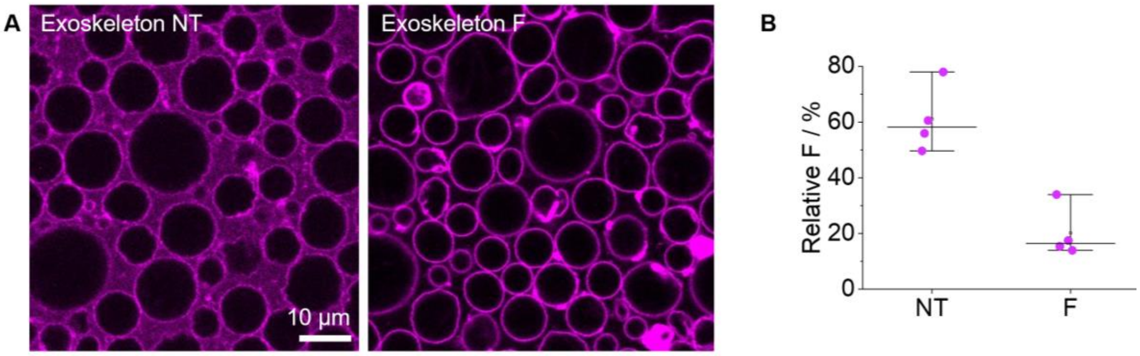

Figure S39. Exploring inter-protocell packing in prototissues. A) CLSM images of protocells coated in either NT or F exoskeletons, scale bar 10 µm. B) Corresponding relative inter-protocell fluorescence derived from A dot plot, calculated by determining the relative membrane fluorescence and percentage inter-protocell gap fluorescence, line and error bars represent the median and standard deviation from 4 measurements.

2.22 Prototissue hyperosmolarity assay

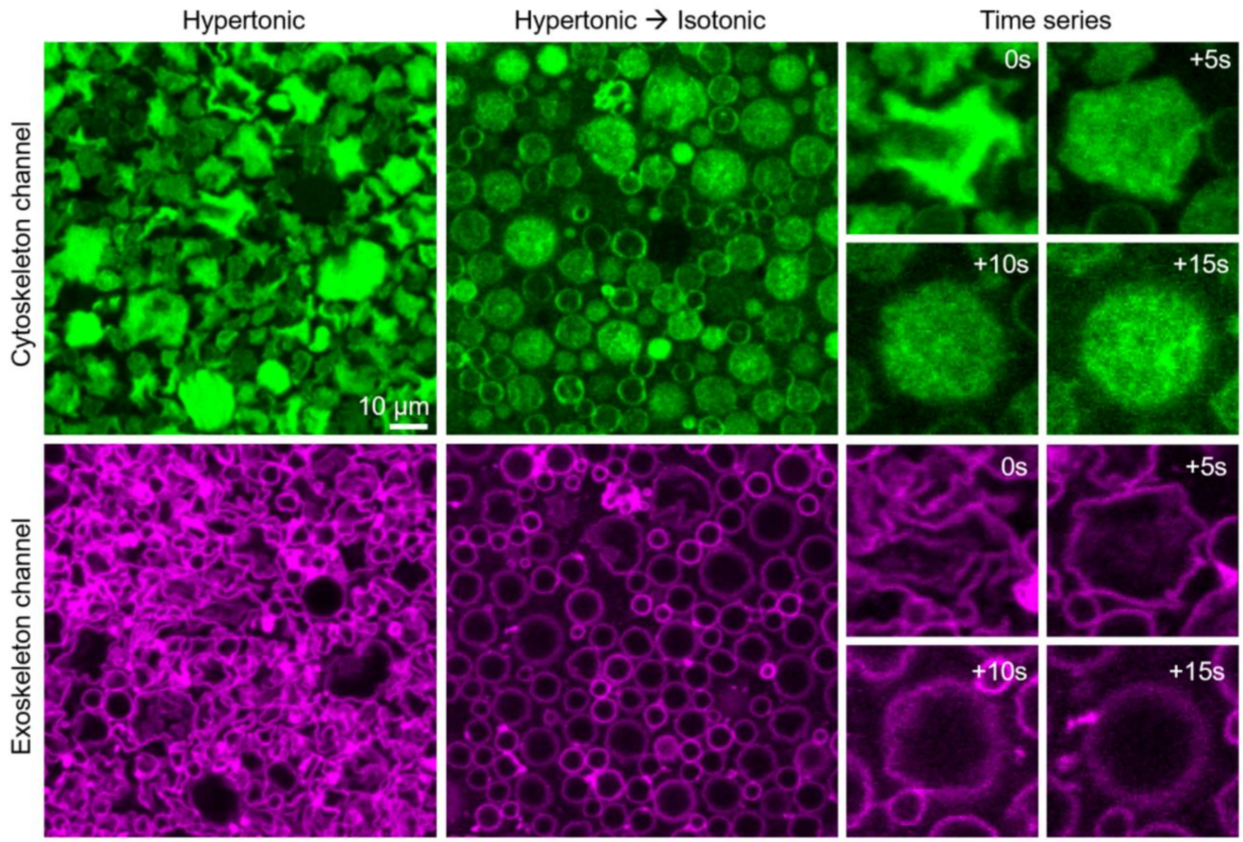

Figure S40. Determining hyperosmolarity stability and reversibility of DMPC : cholesterol : POPG prototissues containing exoskeleton DNA fibers (left column, green channel) and cytoskeleton DNA nanotubes (right column, magenta channel). CLSM images of prototissues under hyperosmotic conditions (~ +400 mOsmol, left column), then returned to isotonic levels by carefully adding water (2.5 µL) to the top of the prototissue droplet (middle column), and time series montage of a magnified protocell in the prototissue at the stated time points after rehydration (right column). Scale bar 10 µm.

2.23 Cross-linking prototissues with external DNA fibers

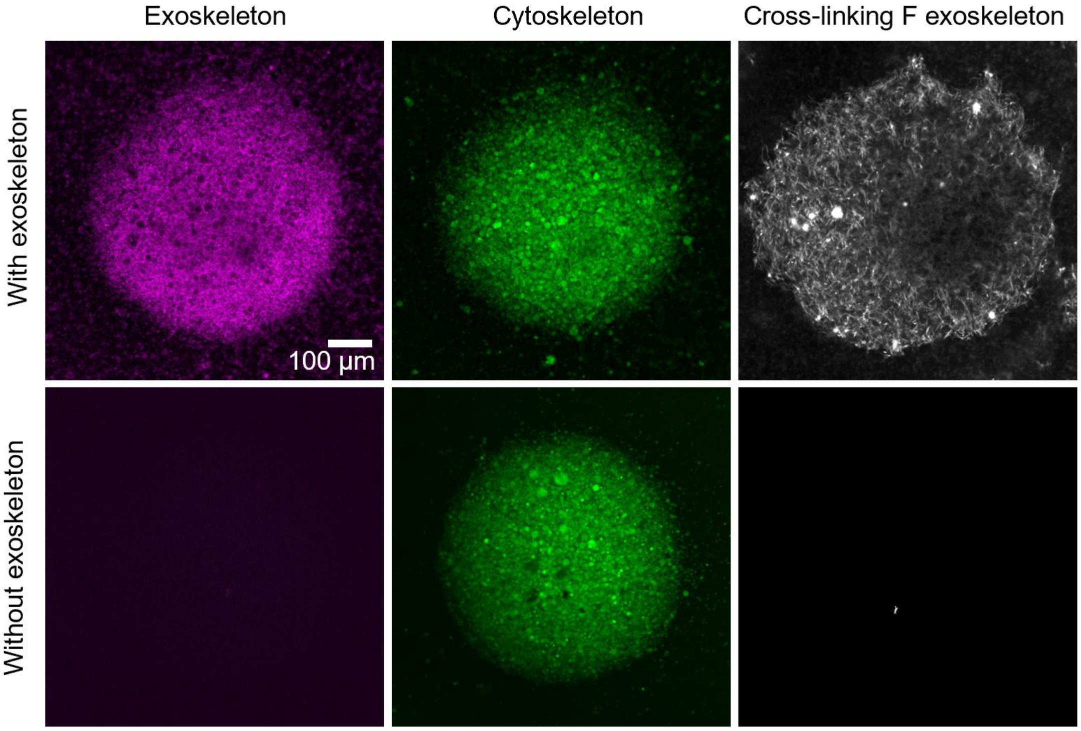

Figure S41. CLSM images of cytoskeleton prototissues (green channel) with (top row) or without (bottom row) exoskeletons (magenta channel) in the presence of external cross-linking DNA fibers (gray channel). External cross-linking fibers selectively colocalised with protocells coated in exoskeletons. Scale bar 100 µm.

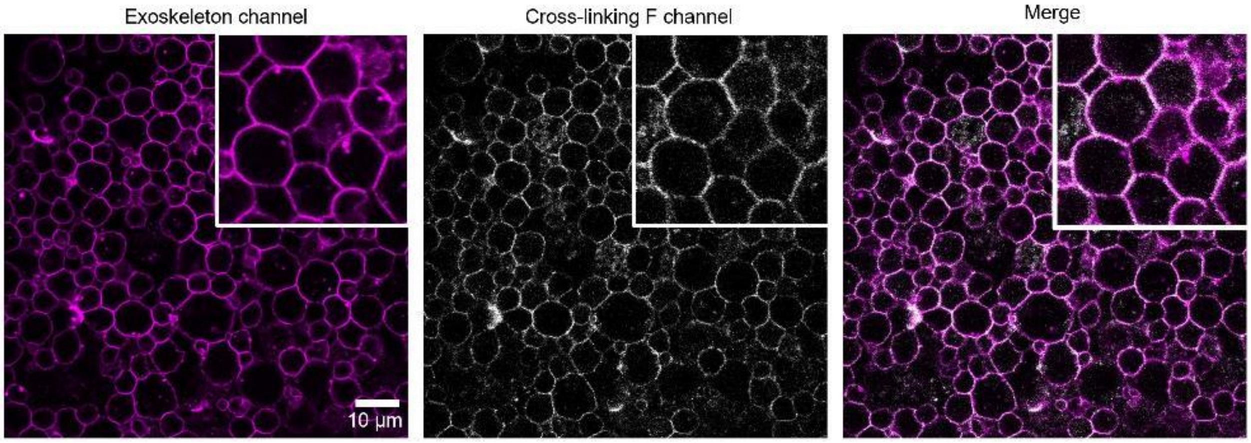

Figure S42. CLSM images of cross-linked protocells in prototissues (magenta channel) mediated via external cross-linking DNA fibers (gray channel). Extensive overlap between exoskeleton protocells and external cross-linking fibers was observed. Scale bar 10 µm.

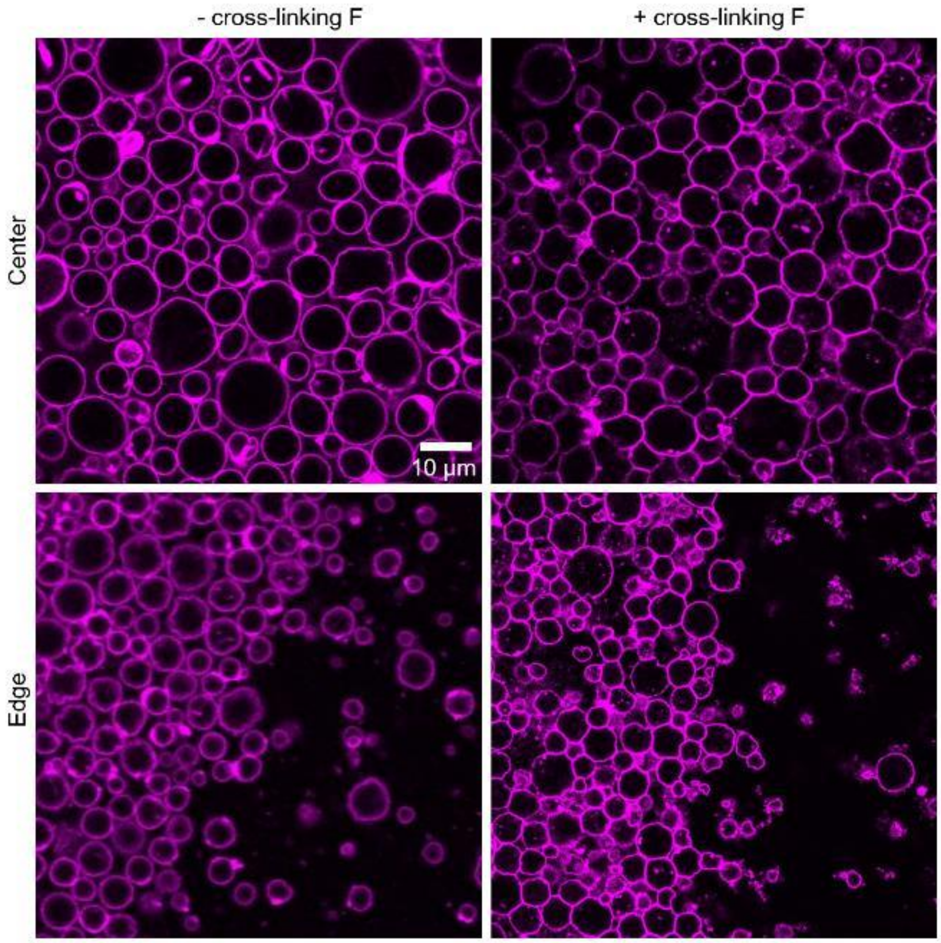

Figure S43. Determining protocell packing in prototissues before and after cross-linking. CLSM images of FAM-labeled DNA fiber exoskeleton prototissues (magenta channel) without (left column) and with (right column) external DNA cross-linking fibers (not shown), center (top row) and edge (bottom row) of prototissue images. Only external cross-linked protocells displayed markedly different protocell packing with well-defined honeycomb-like junctions under these conditions. Scale bar 10 µm.

2.24 Studying prototissue dynamics

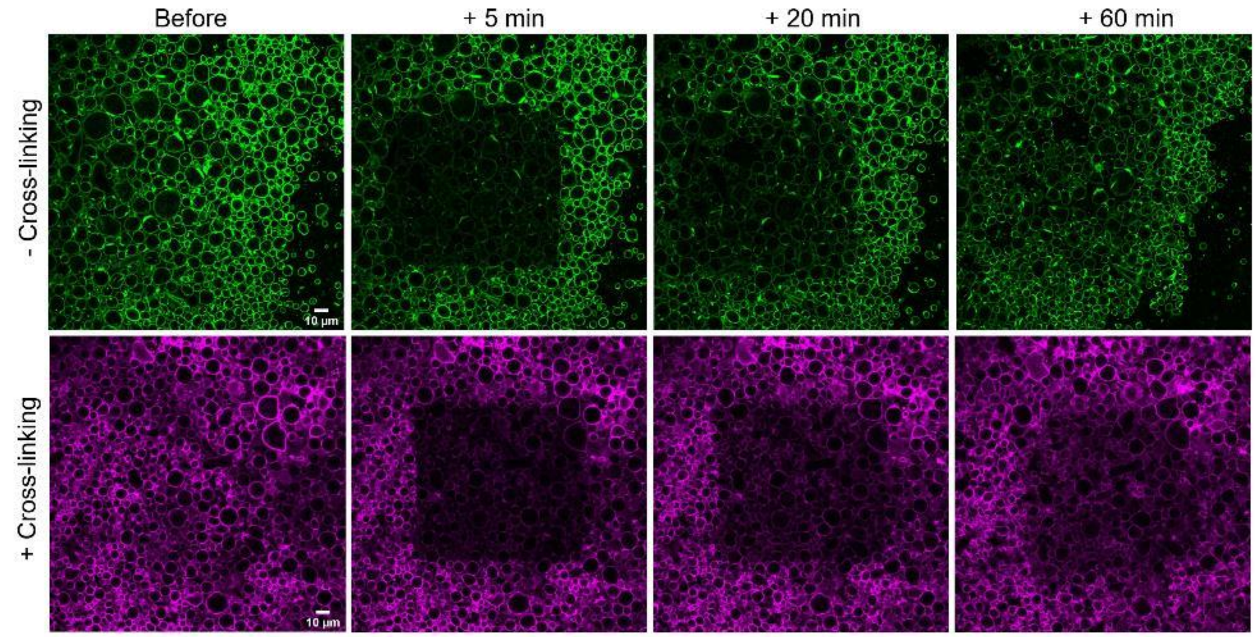

Figure S44. Exploring protocell dynamics in prototissues using FRAP. CLSM images of prototissues at the stated time points before and after FRAP, either without (top row, green channel) or with (bottom row, magenta channel) external cross-linking DNA fibers. The bleached areas displayed well-defined regions after 5 and 20 min, but disappeared for the non-cross linked sample after 60 min. Scale bar 10 µm.

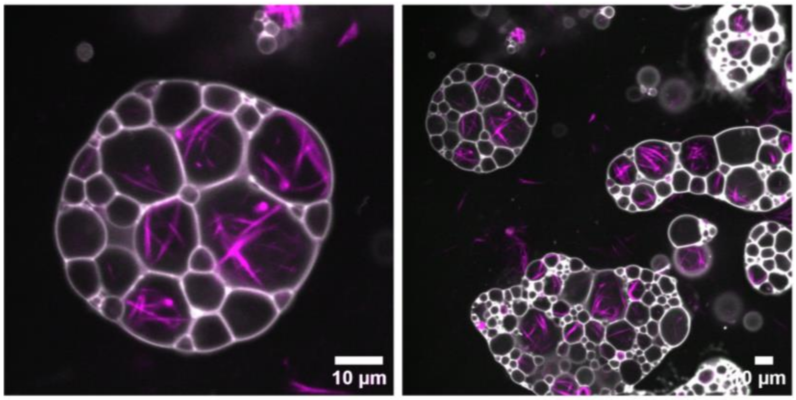

Figure S45. Cross-linked prototissues can be broken down into smaller fragments which are movable. CLSM images of Cy3-labeled 100 mM $MgCl_2$ cytoskeleton DNA fibers (magenta channel) and exoskeleton cross-linking fibers (gray channel) after shearing with a pipettor. Scale bar 10 µm.

2.25 Exploring human blood cell compatiblty and viability

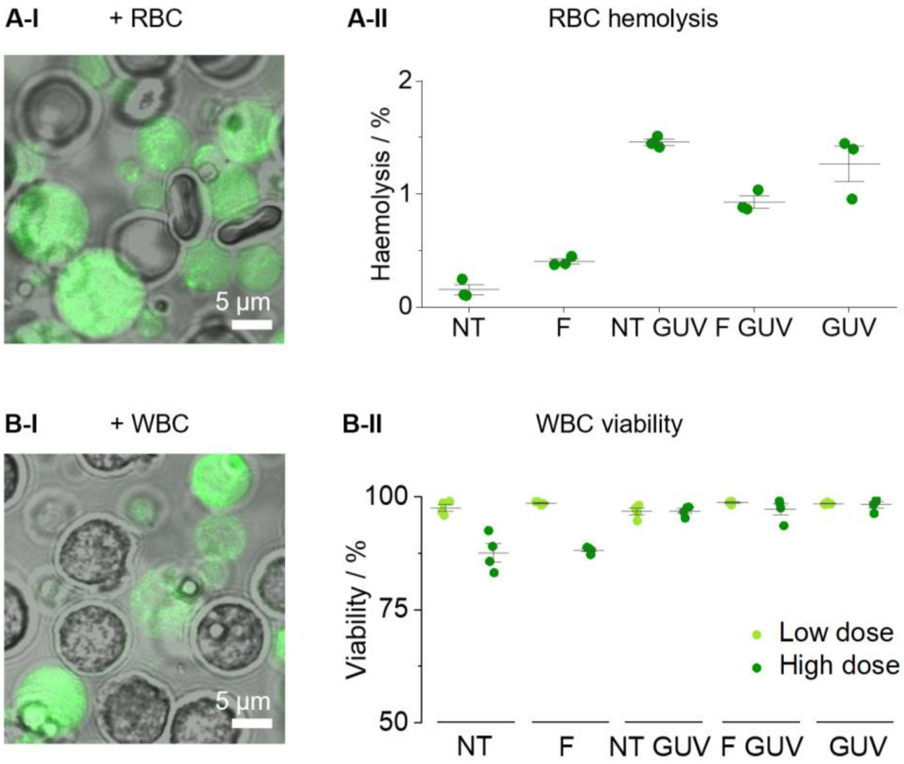

Figure S46. Exploring cytoskeleton DNA protocell biocompatibility with human blood cells. A-I) CLSM images of protocells (green channel) with human erythrocytes (bright field) and A-II) corresponding hemolysis assay dot plot, line and error bars represent the median and standard deviation obtained from 3 independent repeats. B-I) CLSM images of protocells (green channel) with human white blood cells (bright field), and B-II) corresponding FACS derived WBC viability dot plot with low 1 µM (light green) and high doses 10 µM (dark green) of the stated construct. Line and error bars represent the median and standard deviation from 2 individual experiments. Scale bar 5 µm.

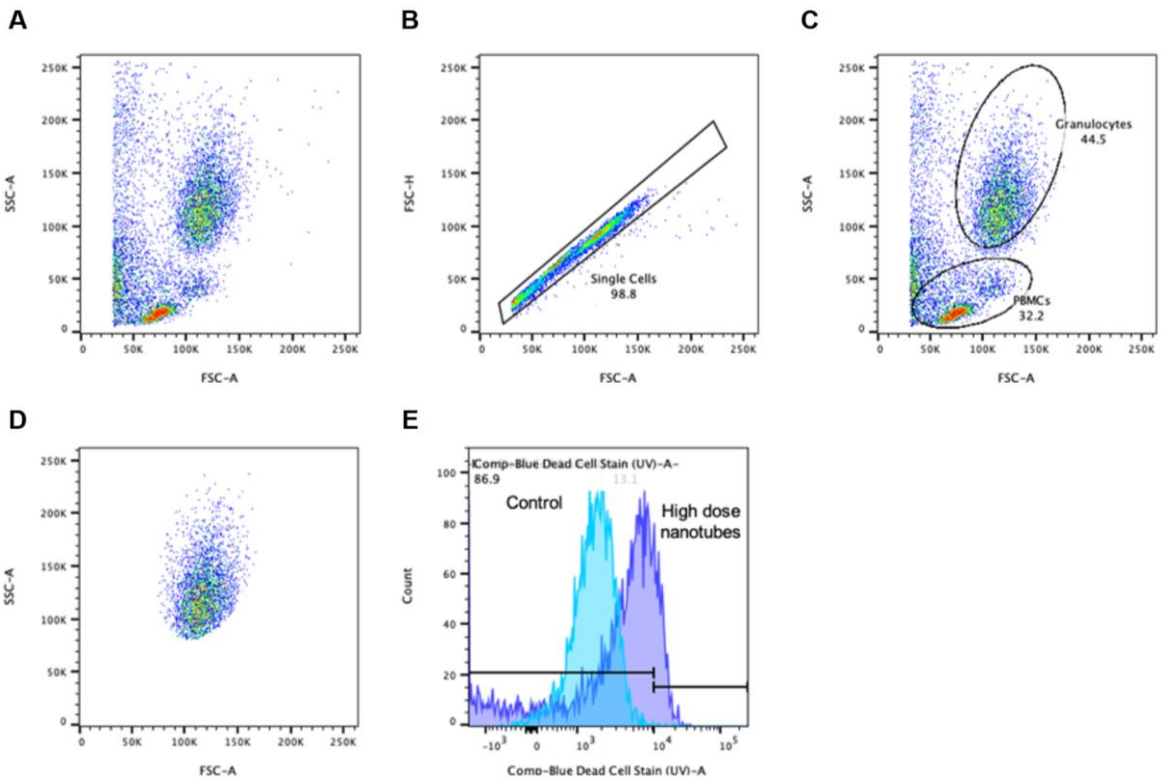

Figure S47. FACS gating strategy. A) Following RBC lysis and live/dead stain, samples were analyzed on flow cytometry. B) Doublets were excluded and single cells used for analysis. C) and D) The two main cell populations, granulocytes and peripheral blood mononuclear cells (PBMCs) were identified by gating on characteristic forward and side scatter profiles. E) Granulocyte were dichotomised into either live or dead cells (using heat-killed cells as a positive control), and dead cells quantified (as percentage of total granulocyte population). Identical gates were applied to all samples.

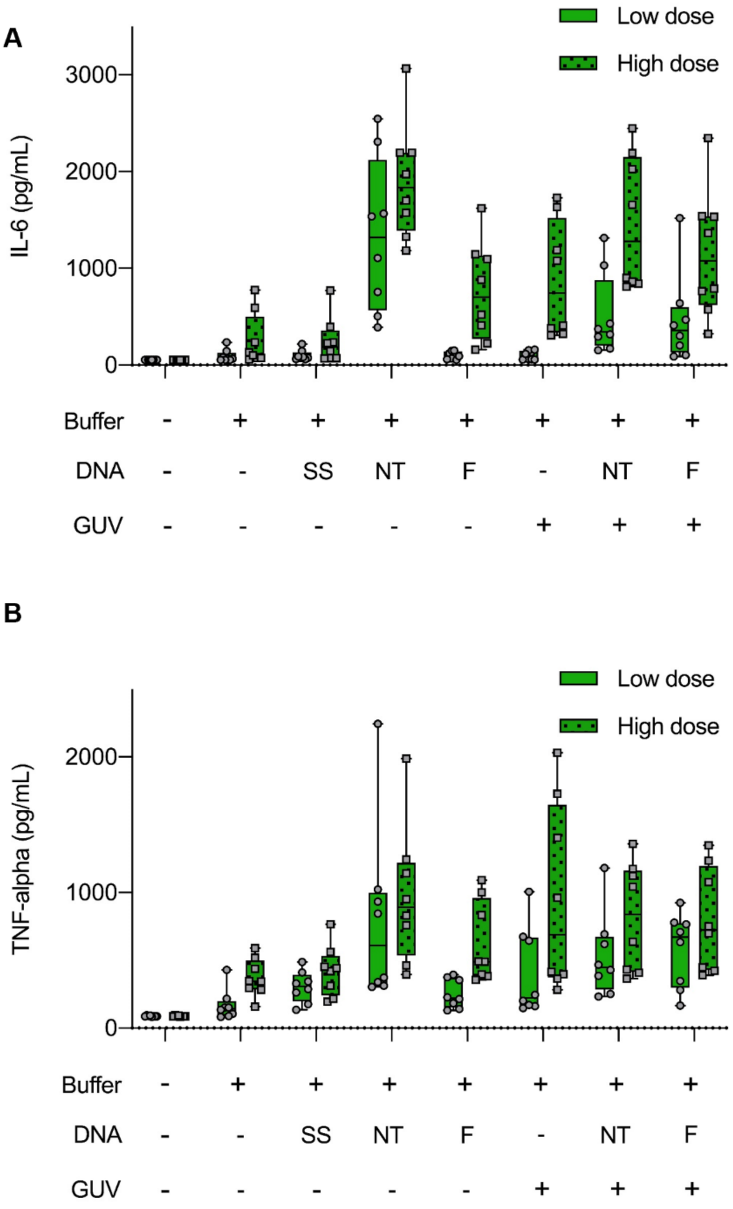

Figure S48. Exploring the effect of DNA nanotubes and fibers at low (1 µM) and high (10 µM) dose, GUVs, and protocells on immune activation as quantified by proinflammatory cytokine A) Interlukin-6 (IL-6) or B) TNF-a production in whole blood. There are differences in inflammation induced by DNA, GUVs, and protocells. Data represent median (bar) and 75th interquartile range (whisker), error bars represent the standard deviation, from 2 experiments.

REFERENCES:

1. Burns, J. R., Introducing bacteria and synthetic biomolecules along engineered DNA fibers. *Small* **2021,** *17*, 11.
2. Sanchez, T.; Chen, D. T.; DeCamp, S. J.; Heymann, M.; Dogic, Z., Spontaneous motion in hierarchically assembled active matter. *Nature* **2012,** *491* (7424), 431-4.
3. Tinevez, J. Y.; Perry, N.; Schindelin, J.; Hoopes, G. M.; Reynolds, G. D.; Laplantine, E.; Bednarek, S. Y.; Shorte, S. L.; Eliceiri, K. W., TrackMate: An open and extensible platform for single-particle tracking. *Methods* **2017,** *115*, 80-90.
