# Supplementary material for: Creating complex protocells and prototissues using simple DNA building blocks
Source: Nat Commun. 2023 Mar 10;14:1314. doi: 10.1038/s41467-023-36875-5 (PMC10006096; doi:10.1038/s41467-023-36875-5)
Supplement: Supplementary file 3 — Reporting Summary [file 41467_2023_36875_MOESM3_ESM.pdf]

nature portfolio

Corresponding author(s): Jonathan R. Burns

Last updated by author(s): 28.01.23

Reporting Summary

Nature Portfolio wishes to improve the reproducibility of the work that we publish. This form provides structure for consistency and transparency in reporting. For further information on Nature Portfolio policies, see our Editorial Policies and the Editorial Policy Checklist.

Statistics

For all statistical analyses, confirm that the following items are present in the figure legend, table legend, main text, or Methods section.

n/a	Confirmed	
☐	☒	The exact sample size (*n*) for each experimental group/condition, given as a discrete number and unit of measurement
☒	☐	A statement on whether measurements were taken from distinct samples or whether the same sample was measured repeatedly
☐	☒	The statistical test(s) used AND whether they are one- or two-sided<br>*Only common tests should be described solely by name; describe more complex techniques in the Methods section.*
☒	☐	A description of all covariates tested
☐	☒	A description of any assumptions or corrections, such as tests of normality and adjustment for multiple comparisons
☐	☒	A full description of the statistical parameters including central tendency (e.g. means) or other basic estimates (e.g. regression coefficient) AND variation (e.g. standard deviation) or associated estimates of uncertainty (e.g. confidence intervals)
☐	☒	For null hypothesis testing, the test statistic (e.g. *F*, *t*, *r*) with confidence intervals, effect sizes, degrees of freedom and *P* value noted<br>*Give P values as exact values whenever suitable.*
☒	☐	For Bayesian analysis, information on the choice of priors and Markov chain Monte Carlo settings
☒	☐	For hierarchical and complex designs, identification of the appropriate level for tests and full reporting of outcomes
☒	☐	Estimates of effect sizes (e.g. Cohen's *d*, Pearson's *r*), indicating how they were calculated

Our web collection on statistics for biologists contains articles on many of the points above.

Software and code

Policy information about availability of computer code

Data collection	CLSM images collected using a Olympus FV-1000 microscope.<br>AFM images collected using a Multimode 8 Bruker atomic force microscope.<br>TEM images collected using a JEM-2100 microscope equipped with a Orius SC200 Gatan camera.<br>WBC were analyzed using flow cytometry on a LSR Fortessa (BD) flow cytometer (BD Biosciences) running.<br>UV melting profiles collected using a Varian Cary 300 Bio UV-vis spectrophotometer.
Data analysis	ImageJ (version 1.53t), Trackmate (version 7.9.2), OriginPro (2022) Prism (5.00) and Excel (2022) software was used to process data and generate graphs. FACS data was processed using BD FACSDiva (version 9) software.

For manuscripts utilizing custom algorithms or software that are central to the research but not yet described in published literature, software must be made available to editors and reviewers. We strongly encourage code deposition in a community repository (e.g. GitHub). See the Nature Portfolio guidelines for submitting code & software for further information.

Data

Policy information about availability of data

All manuscripts must include a data availability statement. This statement should provide the following information, where applicable:

- Accession codes, unique identifiers, or web links for publicly available datasets
- A description of any restrictions on data availability
- For clinical datasets or third party data, please ensure that the statement adheres to our policy

Source data is provided within the Source Data file. All data is available upon reasonable request to the corresponding author.

Human research participants

Policy information about studies involving human research participants and Sex and Gender in Research.

Reporting on sex and gender	For whole cell assays, a total of four participants (healthy volunteers) were included. A small sample size was required to conduct our experiments; which aimed to assess biocompatibility of novel compounds with human blood cells. Recruiting volunteers from both sexes would not have allowed analysis of differences between sexes due to the small sample size. As such we did not recruit healthy volunteers from both sexes; all four healthy volunteers were of the male sex and gender.
Population characteristics	For whole cell assays, a total of four participants (healthy volunteers) were included. A small sample size was required to conduct our experiments; which aimed to assess biocompatibility of novel compounds with human blood cells.
Recruitment	All participants verbally consented and completed a consent form.
Ethics oversight	The study was approved by UCL research ethics committee (REC ref 19181/001).

Note that full information on the approval of the study protocol must also be provided in the manuscript.

Field-specific reporting

Please select the one below that is the best fit for your research. If you are not sure, read the appropriate sections before making your selection.

☒ Life sciences ☐ Behavioural & social sciences ☐ Ecological, evolutionary & environmental sciences

For a reference copy of the document with all sections, see nature.com/documents/nr-reporting-summary-flat.pdf

Life sciences study design

All studies must disclose on these points even when the disclosure is negative.

Sample size	No sample size calculation was performed. The sample size was chosen according to sample size used by previously published reports of similar experiments (n = 3), e.g. Mizuno et. al, Reconstitution of contractile actomyosin rings in vesicles. Nature Communications 2021, 12 (1), 2254 and Agarwal et. al Dynamic self-assembly of compartmentalized DNA nanotubes. Nat Commun 2021, 12 (1), 3557.<br><br>For whole cell assays, a total of four participants (healthy volunteers) were included. A small sample size was required to conduct our experiments; which aimed to assess biocompatibility of novel compounds with human blood cells. Recruiting volunteers from both sexes would not have allowed analysis of differences between sexes due to the small sample size. As such we did not recruit healthy volunteers from both sexes; all four healthy volunteers were of the male sex and gender.
Data exclusions	For protein nanopore dye transport assay, only GUVs which showed active transport were analyzed.<br><br>For diameter, circularity and osmolarity stability assays, objects smaller than 10 micron squared was omitted from the analysis. Only full objects inside the field of view was included in the analysis.<br><br>For prototissue surface area analysis, objects smaller than 10 micron squared was omitted from the analysis. Only full objects inside the field of view was included in the analysis.
Replication	The reproducibility of microscope images is described in the statistics and reproducibility section. The number of counts in the histogram plots is described in the main text and supporting information. The number of independent repeats for dot plots is described in the figure captions.
Randomization	No details of the participants we used in our study.
Blinding	Blinding is not carried out as the prepared samples were analysed by the same person.

Reporting for specific materials, systems and methods

We require information from authors about some types of materials, experimental systems and methods used in many studies. Here, indicate whether each material, system or method listed is relevant to your study. If you are not sure if a list item applies to your research, read the appropriate section before selecting a response.

Materials & experimental systems

n/a	Involved in the study
☒	☐ Antibodies
☒	☐ Eukaryotic cell lines
☒	☐ Palaeontology and archaeology
☒	☐ Animals and other organisms
☒	☐ Clinical data
☒	☐ Dual use research of concern

Methods

n/a	Involved in the study
☒	☐ ChIP-seq
☐	☒ Flow cytometry
☒	☐ MRI-based neuroimaging

Flow Cytometry

Plots

Confirm that:

☒ The axis labels state the marker and fluorochrome used (e.g. CD4-FITC).

☒ The axis scales are clearly visible. Include numbers along axes only for bottom left plot of group (a 'group' is an analysis of identical markers).

☒ All plots are contour plots with outliers or pseudocolor plots.

☒ A numerical value for number of cells or percentage (with statistics) is provided.

Methodology

Sample preparation	Whole blood (5 mL) was collected in heparinized syringes from each healthy donor. Isolated white blood cells (WBCs) were used to assess viability (using flow cytometry). To isolate WBCs, red blood cells (RBCs) were lysed using 1x red cell lysis buffer (BD; Beckton Dickinson biosciences, UK). Cells were washed and re-suspended in 5 mL Hank's buffered saline solution (HBSS). The protocells or DNA constructs (at 1 µM or 10 µM) were added to isolated white blood cell and the samples incubated for 6 hours at 37°C. Cell viability stain (Live/Dead; Thermo Fisher Scientific, UK) was added to cells for 30 min at 37°C and samples assessed using flow cytometry. Heat (65°C for 30 min) was used to kill cells as a positive control and for gating all samples.
Instrument	Cells were analyzed using flow cytometry on a LSR Fortessa (BD) flow cytometer (BD Biosciences) running BD FACSDiva version 9 software.
Software	Flow cytometry data were analysed using FlowJo version 10.0 (Tree Star Inc, USA). Graphs were constructed, and statistical analysis performed using Prism version 9 (GraphPad, (San Diego, USA).
Cell population abundance	Minimum of 5000 events/sample within the granulocyte population (main WBC population) were read. Data was collected from two individual experiments.
Gating strategy	Identical gates were applied to all samples.<br><br>Gating strategies are shown in Figure S47. A) Following red cell lysis and live/dead stain, samples were analysed on flow cytometry. B) Doublets were excluded and single cells used for analysis. C) and D) The two main cell populations, granulocytes and peripheral blood mononuclear cells (PBMCs) were identified by gating on characteristic forward and side scatter profiles. E) Granulocyte were dichotomised into either live or dead cells (using heat-killed cells as a positive control), and dead cells quantified (as percentage of total granulocyte population).

☒ Tick this box to confirm that a figure exemplifying the gating strategy is provided in the Supplementary Information.

March 2021
